# Monoclonal full-length antibody against TAR DNA binding protein 43 reduces related proteinopathy in neurons

**DOI:** 10.1172/jci.insight.140420

**Published:** 2020-11-05

**Authors:** Silvia Pozzi, Philippe Codron, Geneviève Soucy, Laurence Renaud, Pierre Junior Cordeau, Kallol Dutta, Christine Bareil, Jean-Pierre Julien

**Affiliations:** 1 CERVO Brain Research Centre, Québec, Québec, Canada.; 2 UMR CNRS 6015, INSERM U1083, University of Angers, Angers, France.; 3 Department of Psychiatry and Neuroscience, University of Laval, Québec City, Canada.

**Keywords:** Neuroscience, Therapeutics, ALS, Neurodegeneration

## Abstract

Amyotrophic lateral sclerosis (ALS) and frontotemporal lobar degeneration (FTLD), 2 incurable neurodegenerative disorders, share the same pathological hallmark named TDP43 (TAR DNA binding protein 43) proteinopathy. This event is characterized by a consistent cytoplasmic mislocalization and aggregation of the protein TDP43, which loses its physiological properties, leading neurons to death. Antibody-based approaches are now emerging interventions in the field of neurodegenerative disorders. Here, we tested the target specificity, in vivo distribution, and therapeutic efficacy of a monoclonal full-length antibody, named E6, in TDP43-related conditions. We observed that the antibody recognizes specifically the cytoplasmic fraction of TDP43. We demonstrated its ability in targeting large neurons in the spinal cord of mice and in reducing TDP43 mislocalization and NF-κB activation. We also recognized the proteasome as well as the lysosome machineries as possible mechanisms used by the antibody to reduce TDP43 proteinopathy. To our knowledge, this is the first report showing the therapeutic efficacy and feasibility of a full-length antibody against TDP43 in reducing TDP43 proteinopathy in spinal neurons of an ALS/FTLD mouse model.

## Abstract 2

A full-length antibody against TDP43 reduces TDP43 proteinopathy in spinal neurons of an amyotrophic lateral sclerosis/Frontotemporal lobar degeneration mouse model.

## Graphical abstract

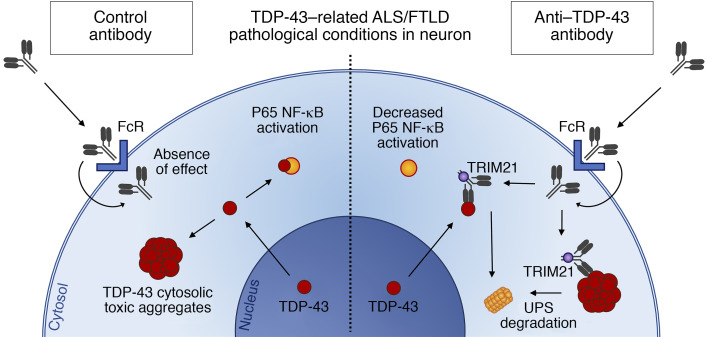

## Introduction

TAR DNA binding protein 43 (also known as TDP43) is a DNA/RNA binding protein predominantly localized in the nucleus of cells (1). Mislocalization and accumulation of hyperphosphorylated, fragmented, and ubiquitinated forms of this protein in the cytoplasm of neurons are known as TDP43 proteinopathy, a pathological hallmark of amyotrophic lateral sclerosis (ALS) and frontotemporal lobar degeneration (FTLD) (2, 3). ALS and FTLD are midlife-onset neurodegenerative disorders, with ALS showing muscle-related symptoms, since the main cells undergoing neurodegeneration are motor neurons (4, 5), and FTLD characterized by changes in the behavior, personality, and/or language, since the cortical prefrontal and temporal neurons are affected (6). Because of TDP43 proteinopathy, ALS and FTLD are now recognized as a disease continuum (7), but the same TDP43 alterations can also be observed in other disorders such as Alzheimer’s disease (AD), Parkinson’s disease (PD), and vascular dementia (1, 8, 9).

Despite different efforts in studying TDP43 during the pathological events, it remains unclear why and how TDP43 mislocalizes and accumulates in the cytoplasm. However, once it starts to accumulate in the cytoplasm, it undoubtedly acquires cytotoxic properties (10). For this reason, therapeutic approaches aimed to reduce cytoplasmic TDP43 accumulation are now being considered (11, 12). In recent years, there has been increasing interest on the use of monoclonal antibodies as a treatment for neurodegenerative disorders (13), with the aim of targeting misfolded intra- or extracellular proteins, such as amyloid β peptide, Tau, or α-synuclein (reviewed in ref. 14). In the field of ALS, immunotherapies against SOD1 protein, involved in familial forms of the disease, have been developed and tested on cellular and mouse models, with encouraging results (15–21).

Recently, we demonstrated the ability of a single-chain antibody against the RRM1 domain of TDP43 to target and reduce cytoplasmic mislocalization/aggregation of TDP43 and to improve motor and cognitive performances in an ALS/FTLD mouse models (22). Having proven the therapeutic efficacy of the antigen binding domain, here we investigated the target recognition, in vivo distribution, and therapeutic potential of the full-length antibody, named E6, against the same RRM1 domain of TDP43.

## Results

### E6 full-length antibody binds specifically to cytoplasmic TDP43.

**Figure 1:**
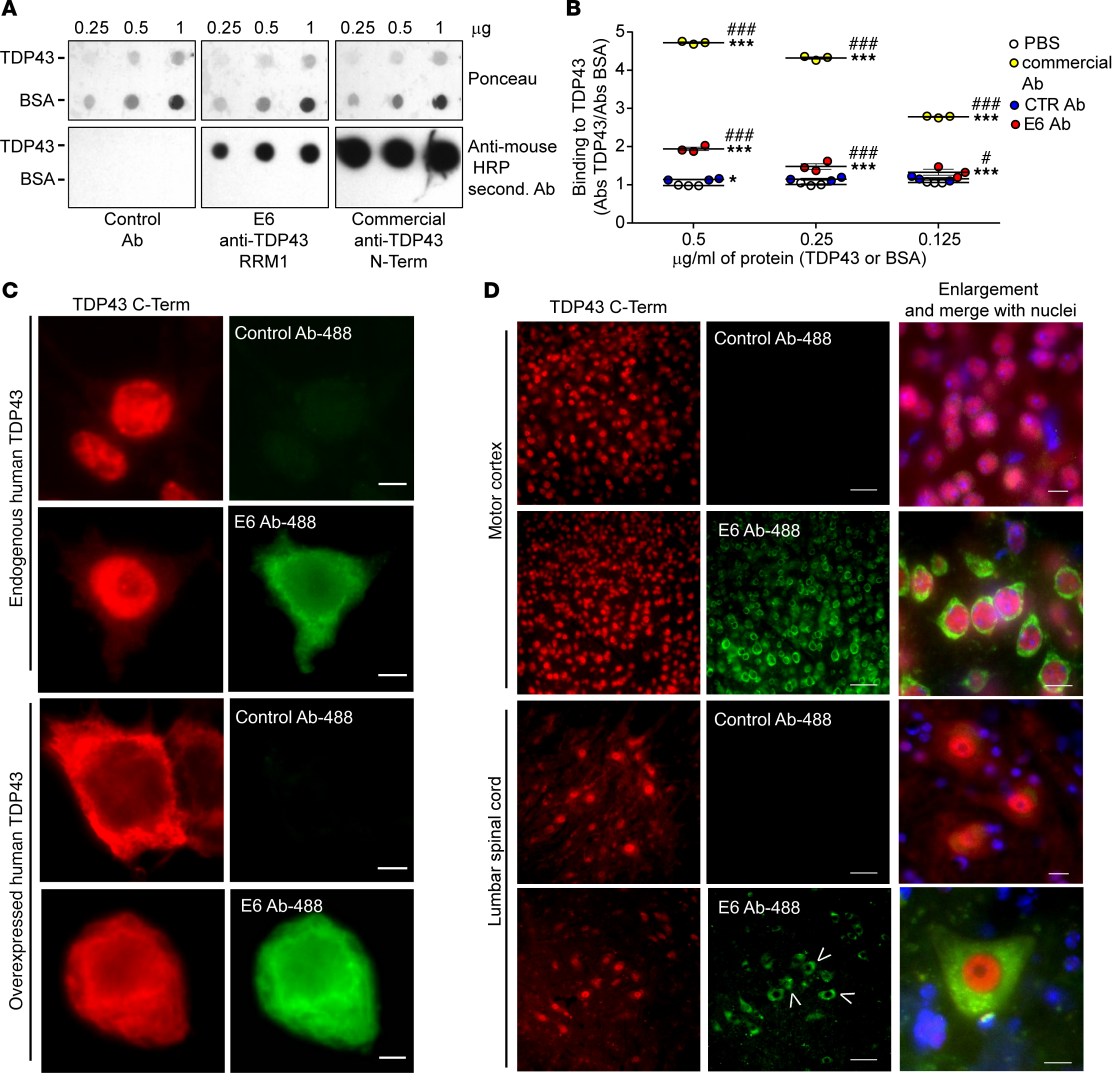
Anti-RRM1 full-length antibody E6 recognizes cytoplasmic localized TDP43 in cells and *TDP43 A315T* mice tissues. (**A**) Different concentrations of human recombinant TDP43 or BSA were loaded on a dot blot membrane and probed with 0.5 μg/mL of control antibody, E6 antibody, or a commercial mouse anti–human TDP43 N-Term antibody (Abnova). Ponceau was used as loading reference. (**B**) ELISA measuring the interaction of 0.4 μg/mL control antibody, E6, or a commercial anti-TDP43 N-Term antibody (Abnova) or PBS on different concentration of TDP43. Data are represented as mean ± SEM; *n* = 3 wells per conditions (dots) and are expressed as ratio of signal (Abs, absorbance) obtained on TDP43 versus BSA. Two-way ANOVA (interaction F_6,24_ = 167.4, *P* < 0.001; concentration F_2,24_ = 256.5, *P* < 0.001; antibody F_3,24_ = 3694, *P* < 0.001), *P < 0.05 or ***P < 0.001 versus PBS, ^#^*P* < 0.05 or ^###^*P* < 0.001 versus control antibody by Tukey’s multiple comparison test. (**C**) Representative immunofluorescence with anti–TDP43 C-Term (Proteintech, red), control-, or E6-488–conjugated (green) on fixed HEK293 cells in standard conditions or after overexpression of *hTDP43-WT*. Scale bar: 5 μm. Merge with nuclei (Hoechst, blue) is shown in Supplemental Figure 1A. (**D**) Representative immunofluorescence with anti–TDP43 C-Term (Proteintech, red), control-, or E6-488–conjugated antibodies (green) and merge with nuclei (Hoechst, blue) on motor cortex or lumbar spinal cord of 10-month-old *TDP43A315T* mice. Scale bar: 50 μm and 10 μm in the enlarged (5×) pictures. Spot-like staining is marked by arrowheads. Merge with nuclei in nonenlarged pictures is shown in Supplemental Figure 1C. Immunofluorescence was performed in more than 3 replicates for **C** and in 3 mice for **D**. The control antibody used was clone 807.33.

We already knew that the E6 monoclonal antibody recognized TDP43 in nuclear cell lysates by Western blot (22). Here, we compared the binding property of the E6 mouse IgG2A anti-RRM1 TDP43 antibody with a commercial mouse anti–human TDP43 N-Term (Abnova). As demonstrated by dot blot analysis (Figure 1A) and ELISA (Figure 1B), the monoclonal anti-RRM1 antibody specifically binds human TDP43 without cross-reactivity to BSA. Interestingly, we observed that, in both assays, E6 antibody showed lower binding affinity for TDP43 than the commercial antibody. A mouse monoclonal IgG2A antibody generated against the G1 glycoprotein of La Crosse Virus (clone 807.33) was used as a control, and it did not show any reactivity against TDP43.

To better understand the features of E6 antibody target recognition, we investigated the specificity of binding to TDP43 by immunofluorescence microscopy (Figure 1, C and D). To avoid antigen binding competition effects and secondary antibody–aspecific signals, we decided to localize TDP43 with a commercial polyclonal anti–C-Term TDP43 (Proteintech) and to label the E6 anti–RRM1 TDP43 or the control mouse IgG2A with a fluorescent dye (Alexa Fluor 488). We first performed experiments on human HEK293 cells (Figure 1C and Supplemental Figure 1A; supplemental material available online with this article; https://doi.org/10.1172/jci.insight.140420DS1) in conditions where the endogenous TDP43 is mainly localized in the nucleus or in the presence of human TDP43 overexpression, which provokes an aberrant mislocalization of TDP43 protein in the cytoplasm. It is noteworthy that the E6 anti-RRM1 antibody primarily detected the cytoplasmic form of TDP43 in contrast with the commercial antibody, which mainly detected nuclear TDP43 with only a faint signal of cytoplasmic TDP43. No immunoreactivity was observed with the fluorescein-labeled control antibody 807.33. On the same cells, we performed Western blot analyses (Supplemental Figure 1B), and we observed that, in total extract of cells, the E6 antibody mainly recognized the 35KDa protein, which is one of the fragmented and pathological forms of the protein found in the cytoplasm (23).

We then tested the anti–RRM1 TDP43 E6 antibody on tissues from 10-month-old mice overexpressing human mutant *TDP43A315T*, a mouse model that shows histopathological and phenotypical features of ALS/FTLD pathology such as TDP43 proteinopathy (24) (Figure 1D and Supplemental Figure 1C). The E6 antibody, but not the control antibody, detected the cytoplasmic mislocalized TDP43 in both cortical and spinal neurons, 2 regions of the CNS affected by the disease. E6 was also able to detect spot-like staining in the cytoplasm of cells — presumably TDP43 aggregates. Interestingly, as observed in Hek293 cells with endogenous expression of TDP43, the E6 antibody was able to detect the physiological cytoplasmic TDP43 present in cells also in motor cortical neurons and large neurons of the spinal cord in nontransgenic mice (Supplemental Figure 1D).

**Figure 2:**
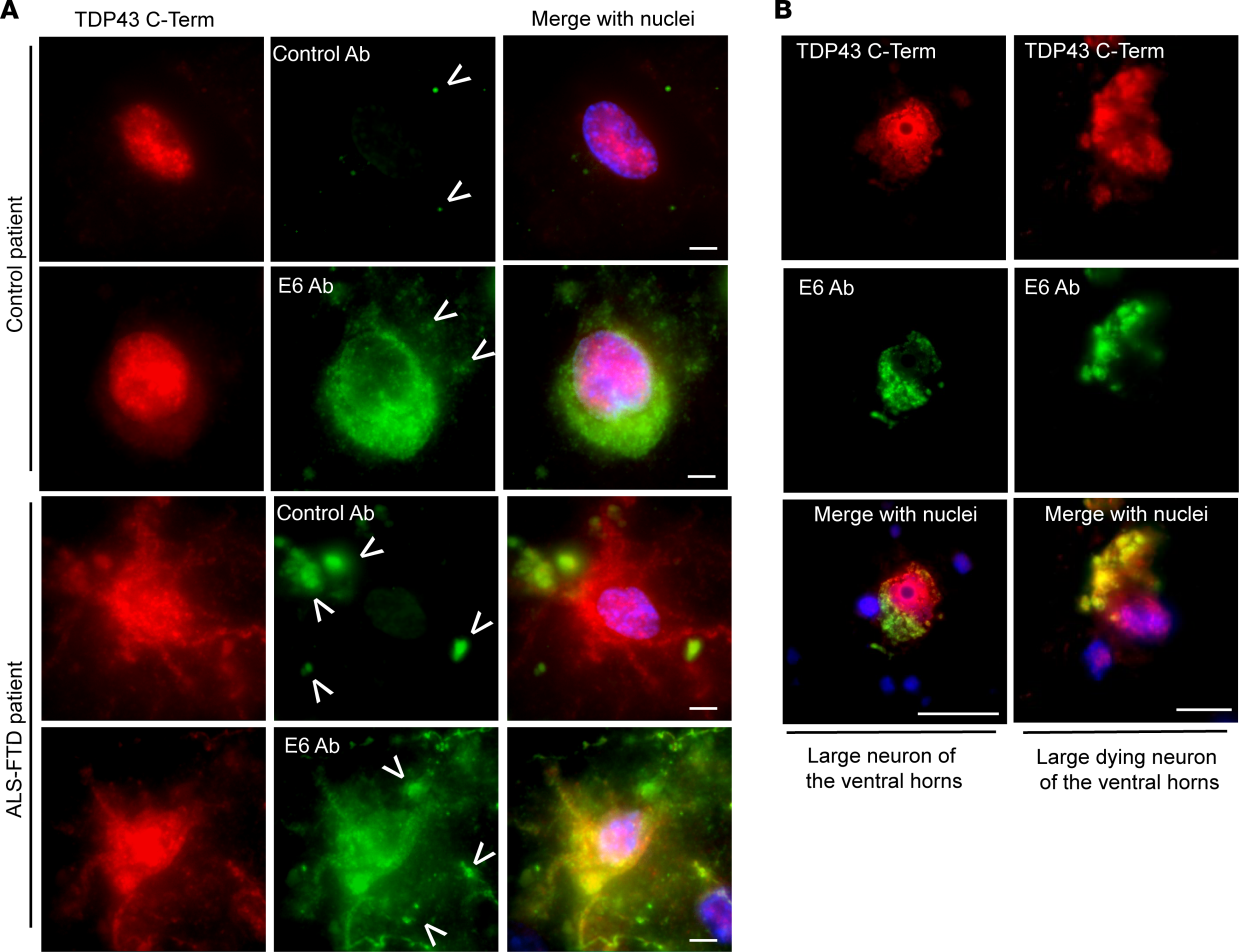
Anti-RRM1 full-length antibody E6 recognizes cytoplasmic localized TDP43 in cortical and spinal cord tissues of ALS/FTD patients. (**A**) Representative immunofluorescence with anti–TDP43 C-Term (Proteintech, red), unlabeled control, or E6 antibodies (green) and merge with nuclei (Hoechst, blue) on prefrontal cortex of a control nonneurodegenerative patient and a FTLD patient. Scale bar: 5 μm. Lipofuscin nonspecific spots were visible in all fluorescence channels and are marked as arrowheads. (**B**) Representative immunofluorescence with anti–TDP43 C-Term (Proteintech, red), unlabeled E6 antibody (green), and merge with nuclei (Hoechst, blue) on spinal cord ventral horns of an ALS patient. Scale bar: 10 μm.

We also evaluated the binding specificity of the E6 antibody with human brain and spinal cord samples (Figure 2). Prefrontal cortices of control and a TDP43-related FTLD patient samples were probed with unlabeled E6 and control antibody 807.33. Mislocalization of TDP43 was previously confirmed on tissues by IHC for TDP43 (Supplemental Figure 1E). E6 recognized cytoplasmic TDP43 in both control and FTLD patient tissues, confirming its specificity for the cytosolic form of TDP43 under physiological and pathological conditions. Moreover, in FTLD tissue sections, the cytoplasmic TDP43 aggregates were detected with both E6 and the commercial anti–TDP43 C-Term antibody (Figure 2A). Finally, we performed immunofluorescence staining in spinal cord samples of an ALS patient (Figure 2B), where we demonstrated the peculiar colocalization of E6 with only the cytoplasmic TDP43 in large neurons of the ventral horns. Also in this pathological tissue, it was evident how E6 could stain for both diffuse and aggregated forms of TDP43.

### E6 full-length antibody is internalized in cells.

**Figure 3:**
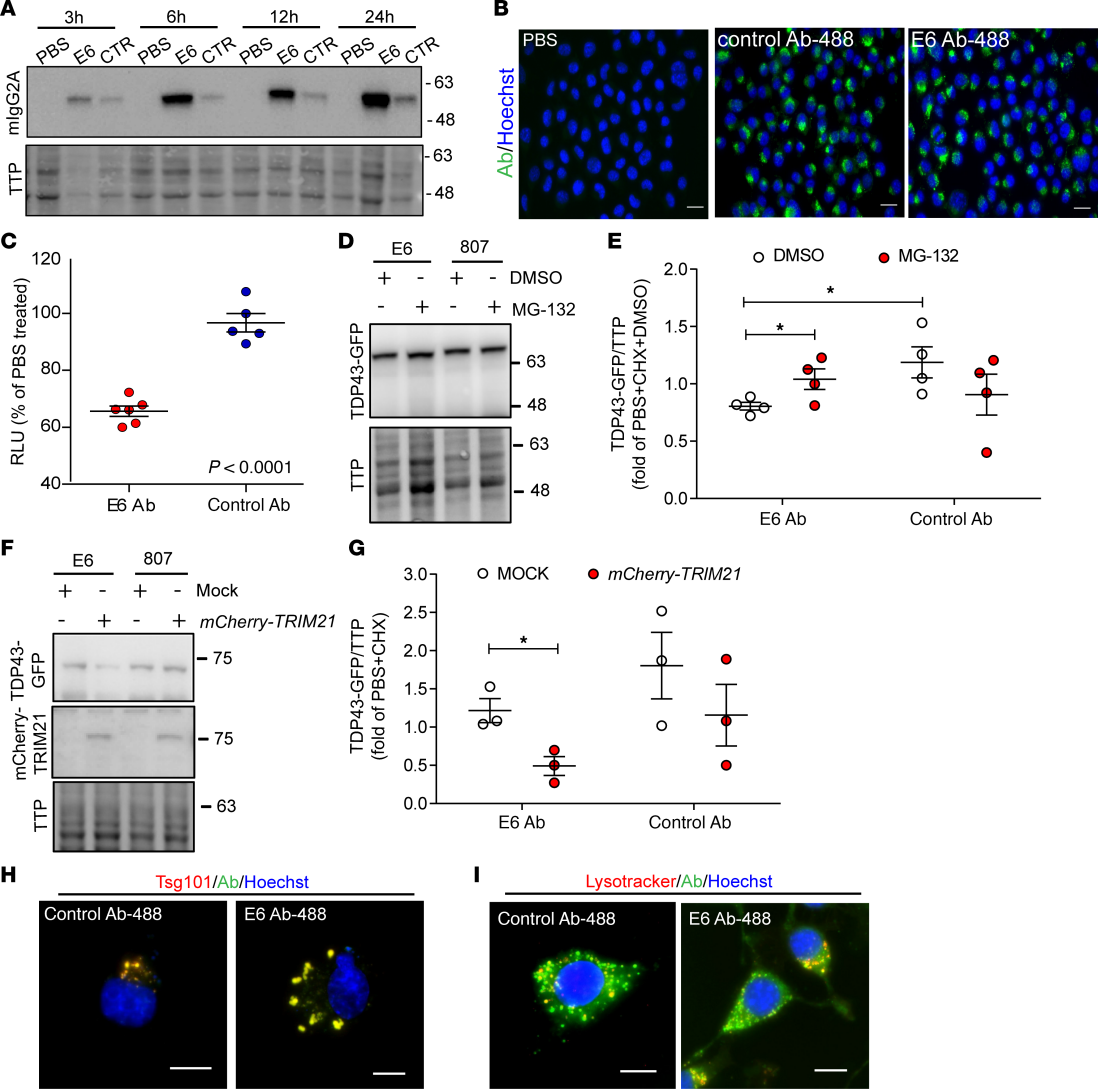
E6 full-length antibody is internalized in culture cells and reduces NF-κB activation and cytoplasmic TDP43 in vitro. (**A**) Representative Western blot showing heavy light chain (50 kDa) form control- or E6 antibodies in total lysates of neuronal N2A cells after cycloheximide (CHX) and 10 μg/mL E6 or control antibody treatments. Total transferred proteins (TTP) were considered as loading reference. Experiment was replicated more than 3 times. (**B**) Representative fluorescence microscopy images showing control- or E6-488–conjugated antibodies (green) internalized in the cytoplasm of neuronal N2A cells after 24 hours of treatment. Nuclei are labeled with Hoechst (blue). Scale bar: 20 μm. Images were acquired with automatic light intensity regulation. Single channel for antibody-conjugated signal is shown in Supplemental Figure 2A. (**C**) NF-κB activation was evaluated in BV2-*p65-luc* cells by measuring the luciferase activity (RLU, relative luminescence units) after treatment. Data are represented as mean ± SEM; *n* = 5–6 replicates from 3 independent experiments (dots) and are expressed as fold of PBS-treated, unpaired *t* test analysis. (**D** and **E**) Representative Western blot (**D**) and quantification (**E**) of the overexpressed protein (70 kDa) in the purified cytoplasmic fraction of treated cells; total transferred proteins (TTP) were considered as loading reference. Data are represented as mean ± SEM; *n* = 4 independent experiments (dots) and are expressed as fold of cells treated with PBS, CHX, and DMSO. Two-way ANOVA (interaction F_1,2_ = 4.496, *P* = 0.0555; treatment F_1,2_ = 1.032, *P* = 0.3298; Ab treatment, F_1,2_ = 0.0346; *P* = 0.8554). *P < 0.05 by unpaired *t* test analysis. (**F** and **G**) Representative Western blot (**F**) and quantification (**G**) of the overexpressed protein (70 kDa) in the purified cytoplasmic fraction of treated cells; total transferred proteins (TTP) were considered as loading reference. Data are represented as mean ± SEM; *n* = 3 independent experiments (dots) and are expressed as fold of cells treated with PBS and CHX. Two-way ANOVA (interaction F_1,8_ = 0.0163, *P* = 0.9014; treatment F_1,8_ = 4.015, *P* = 0.0800; Ab treatment F_1,8_ = 4.821, *P* = 0.0594). *P < 0.05 by unpaired *t* test analysis. (**H** and **I**) Representative merged channels of fluorescence microscopy images showing the colocalization of 488-conjugated antibodies (green) with Tsg101 (red) late endosomal marker (**H**) or lysotracker (red) (**I**) in neuronal N2A cells treated 24 hours with 10 μg/mL of antibodies. Scale bar: 20 μm. Images were acquired with automatic light intensity regulation. Single channels for the antibodies, Tsg101, or lysotracker signals are shown in Supplemental Figure 3, F and G. Experiments showed in panels H and I were repeated at least 3 times. The control antibody used was clone 807.33.

One of the major problems in working with full-length antibodies is their low capacity to cross the cellular membrane and target intracellular proteins. The ability of the E6 antibody to penetrate in living cells was investigated by treating mouse neuroblastoma cells (Neuro2A; N2A) with E6 or control antibody. We exposed cells to 10 μg/mL of antibodies and verified the presence of the antibody intracellularly by Western blot at different time points (Figure 3A). The E6 antibody penetrated cells, and internalization was detected after 3 hours of treatment. The amount of internalized antibodies increased with time. We also confirmed the ability of the antibody to penetrate the cells and localize mainly in the cytoplasm by immunofluorescence on cells treated for 24 hours with labeled E6 or control antibodies (Figure 3B and Supplemental Figure 2A). Internalization of E6 or control antibodies did not induce cellular death (Supplemental Figure 2B). We finally verified the internalization of the antibody in N2A cells transfected for 24 hours with human *mNLS-TDP43*, which — by having a mutation inside the nuclear localization signal — allows a significant cytoplasmic distribution of the TDP43 protein (25). The E6 monoclonal antibody, as opposed to the control, was detectable inside the cell soon after 30 minutes from treatment, and its presence increased with the time of exposure (Supplemental Figure 2C).

### E6 antibody reduces cytoplasmic TDP43 and NF-κB activation in cultured cells.

We previously demonstrated that E6 antibody is able to disrupt the interaction between human recombinant TDP43 and p65, the main subunit of NF-κB (22). Moreover, the single-chain antibody derived from E6 had demonstrated the ability to reduce NF-κB activation in microglial cells both in vitro and in vivo. We therefore evaluated the ability of the E6 antibody to reduce NF-κB activation in a well-established cellular model, the BV2-*p65-luc* microglial cells (26) challenged with LPS. We treated cells with 2.5 μg/mL of antibody and then activated them for 4 hours with 500 ng/mL of LPS, for a total of a 6-hour treatment with the antibody (Supplemental Figure 3A). After treatment, we confirmed the presence of the antibodies inside microglial cells (Supplemental Figure 3B), and we observed that the E6 anti-TDP43 antibody reduced by about 35% the activation of NF-κB in microglial cells after LPS challenge (Figure 3C).

The previously studied E6-derived single-chain antibody was found to reduce the amount of cytoplasmic TDP43 by enhancing ubiquitination of the protein toward the proteasome and autophagic degradative pathways (22). The Fc domain of the E6 full-length antibody may confer an advantage in boosting degradation of the protein target. It has indeed been demonstrated that internalized antibodies can bind to the Fc intracellular receptor TRIM21, which drives the antibody-target complex to the proteasomal degradation (27–29). We therefore examined if E6 can mediate TDP43 degradation by the TRIM21-dependent mechanism in N2A cells transfected with *GFP-mNLS-TDP43* and treated with 10 μg/mL of E6 antibody (Supplemental Figure 3C). Indeed, E6 antibody was able to reduce the amount of cytoplasmic TDP43 compared with the control antibody (Figure 3, D and E) by about 33%. Interestingly, by inhibiting the proteasome by MG-132 treatment, we noticed TDP43 degradation impairments only in the presence of E6, suggesting the involvement of this cellular component in the E6-mediated TDP43 degradation.

We also evaluated cytoplasmic TDP43 degradation in N2A *GFP-mNLS-TDP43*–expressing cells cotransfected with *mCherry-TRIM21* (Supplemental Figure 3D). By overexpressing TRIM21, we observed a 59% significant reduction of TDP43 only in cells treated with E6 antibody (Figure 3, F and G), confirming the involvement of TRIM21 in the degradation of TDP43 when E6 antibody is present.

Finally, by analyzing the E6 localization in treated cells at higher magnification, we noticed both a diffuse and a spot-like staining of the penetrating antibody (Supplemental Figure 2A). We therefore determined whether an antibody could be internalized also in degradative vesicles (30). We found that both E6 and control antibodies, after 24 hours of treatment, localized in late endosomes (Figure 3H and Supplemental Figure 3F) and lysosomes (Figure 3I and Supplemental Figure 3G), supporting the idea that a small portion of an antibody inside the cell, and potentially its antigen, could be lately degraded also through the lysosomal pathway.

### Tissue and cellular distribution of the E6 antibody after intranasal, i.p., intracerebroventricular, and intrathecal injections.

**Figure 4:**
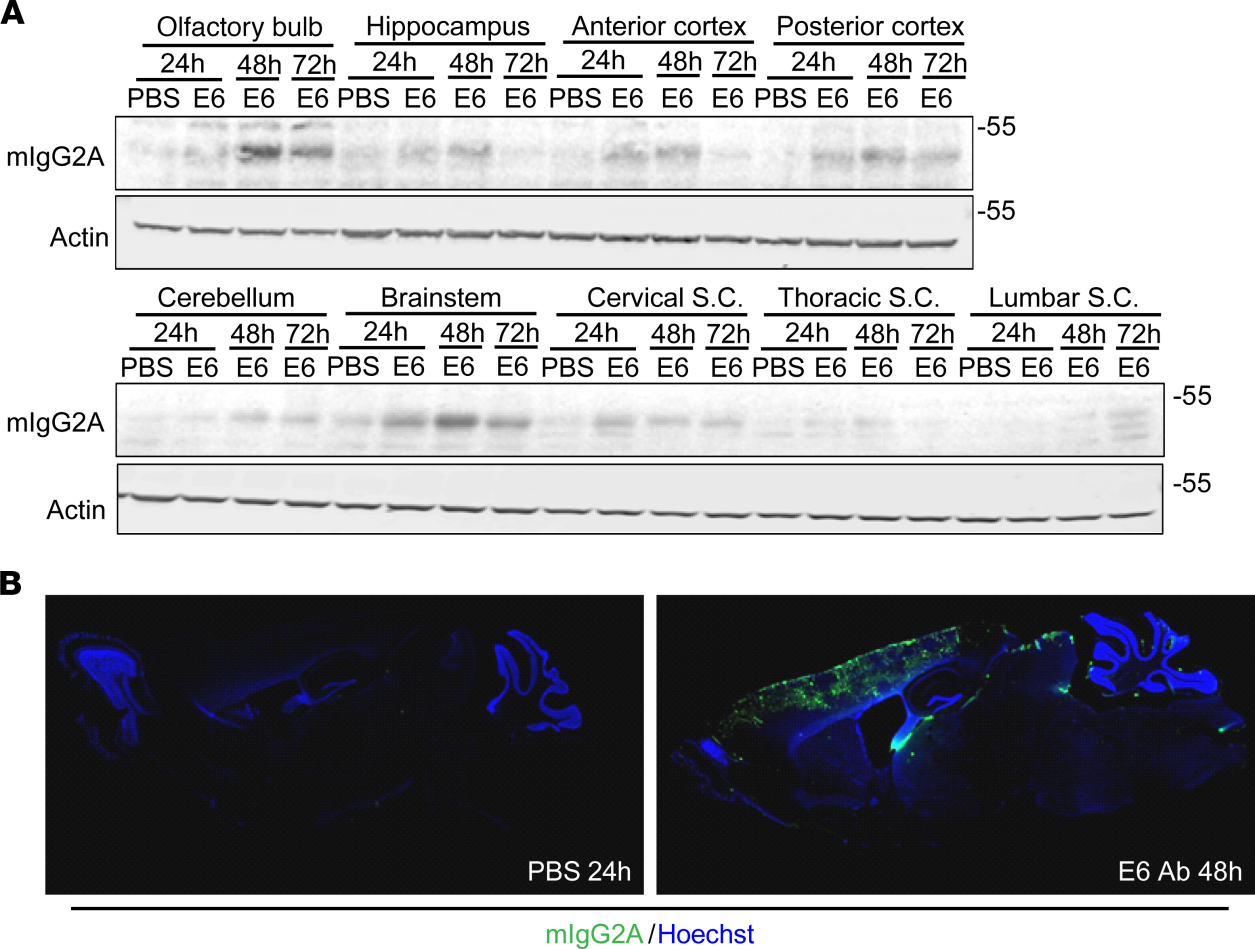
Tissue and cellular distribution after intranasal delivery. (**A**) Western blot for mIgG2A and actin on total lysate from different brain and spinal cord regions of mice treated intranasally with E6 Ab or equal volume of PBS. (**B**) E6 Ab distribution in sagittal sections of brain. Mice that were PBS treated were sacrificed after 24 hours, whereas E6 Ab–treated mice were sacrificed after 48 hours. Pictures represent merged signal from mIgG2A (green) and nuclei (Hoechst, blue). A 2.5× enlargement is shown. One mouse per condition was used, but results were confirmed in multiple sections from the areas of interest.

**Figure 5:**
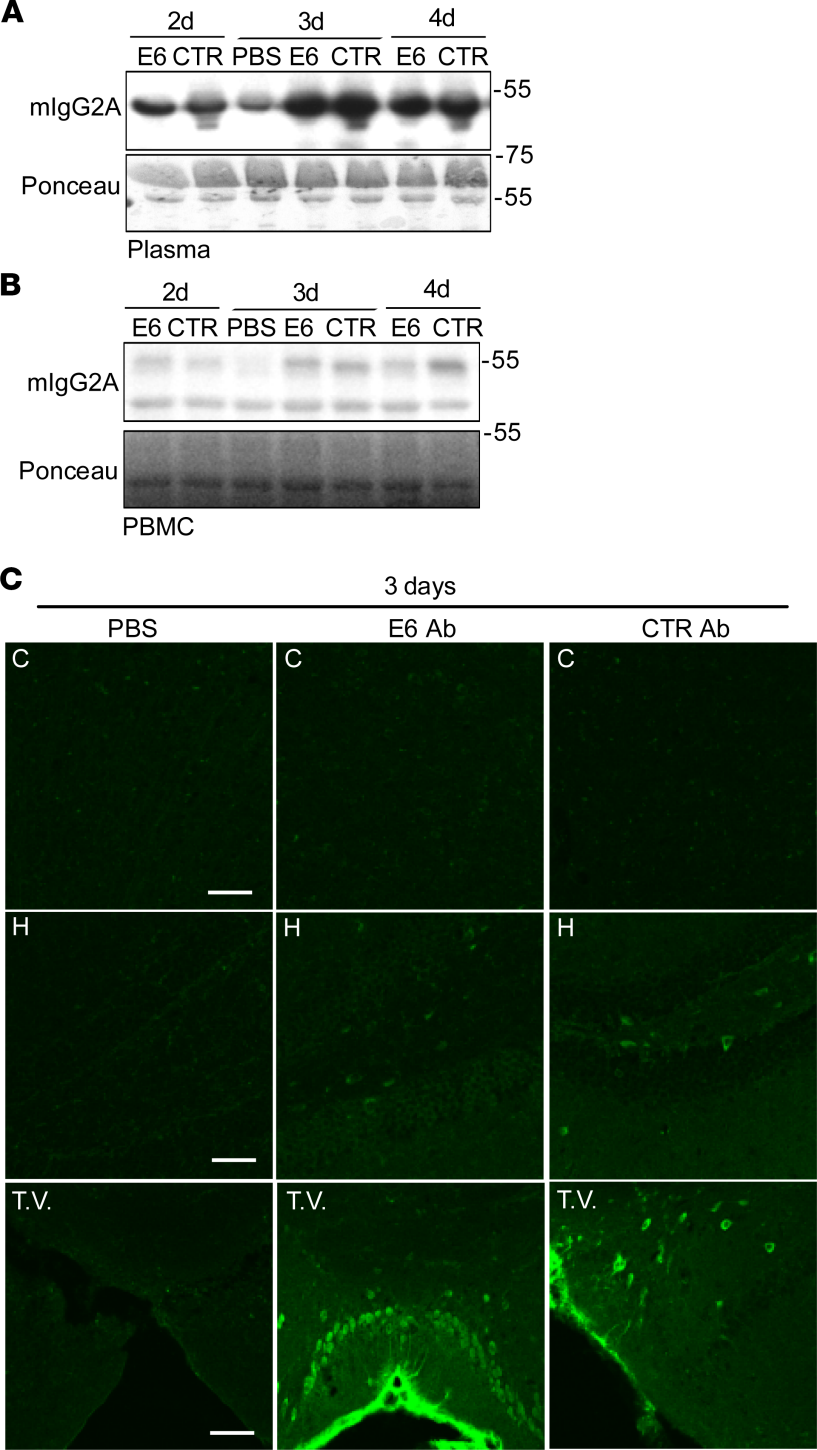
Tissue and cellular distribution after i.p. delivery. (**A** and **B**) Western blot for mIgG2A in plasma (**A**) and PBMC (**B**) total lysate from mice injected i.p. with E6, CTR Ab, or equal volume of PBS. Ponceau was used as loading control. (**C**) Ab distribution after 3 days from injection in coronal sections of cortex (C), hippocampus (H), and third ventricle (T.V.). Signal represents mIgG2A (green). Scale bar: 50 μm. One mouse per condition was used, but results were confirmed in multiple sections from the areas of interest. The control antibody used was clone 807.33.

**Figure 6:**
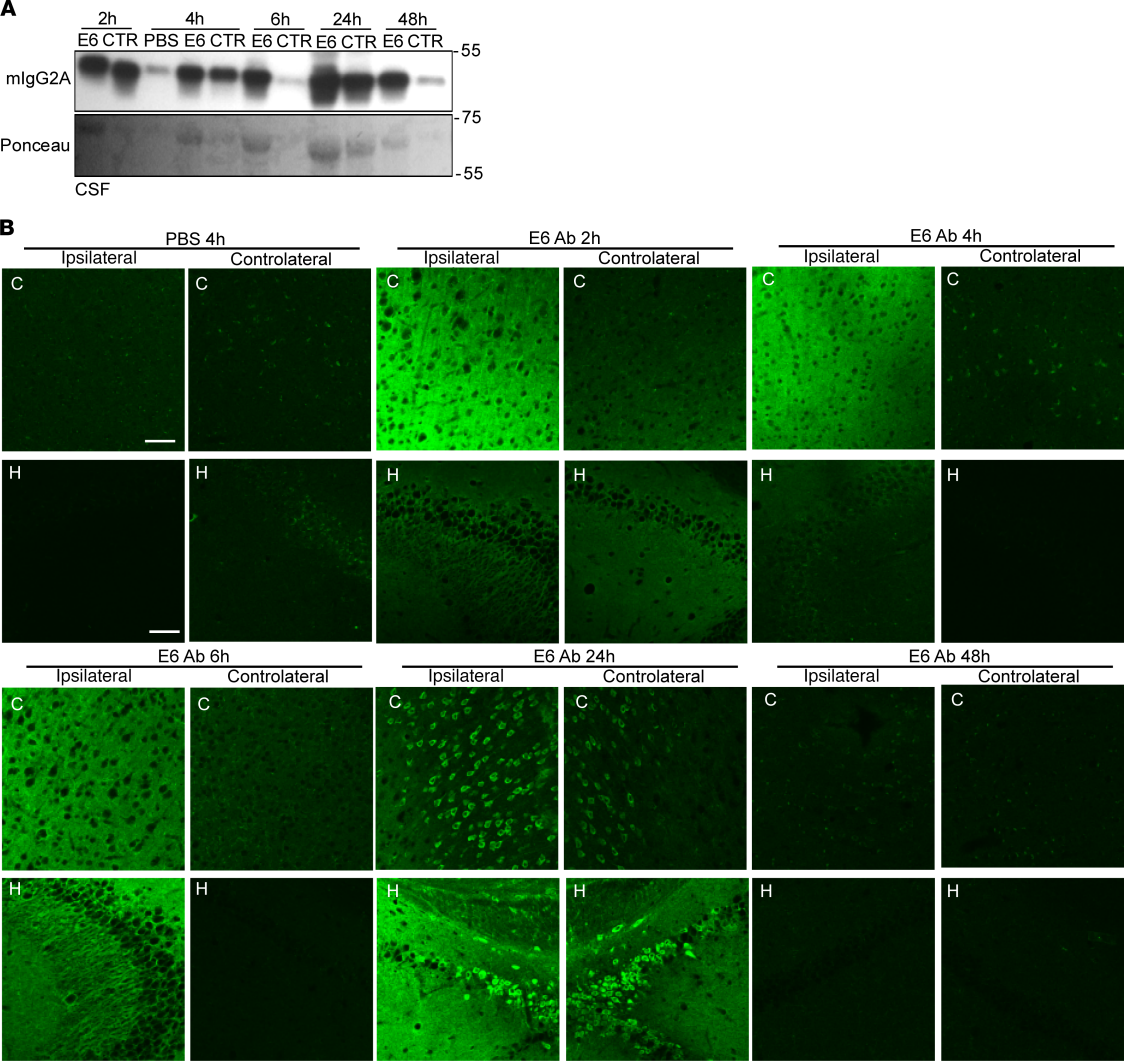
Tissue and cellular distribution after intracerebroventricular (ICV) delivery. (**A**) Western blot for mIgG2A in CSF from mice injected intracerebroventricularly with E6, CTR Ab or equal volume of PBS. Ponceau was used as loading control. (**B**) E6 Ab distribution in coronal sections of cortex (C) and hippocampus (H). Ipsilateral and contralateral sections have been both considered. Signal represents mIgG2A (green). Scale bar = 50 μm. One mouse per condition was used but results were confirmed in multiple sections from the areas of interest. The control antibody used was clone 807.33.

**Figure 7:**
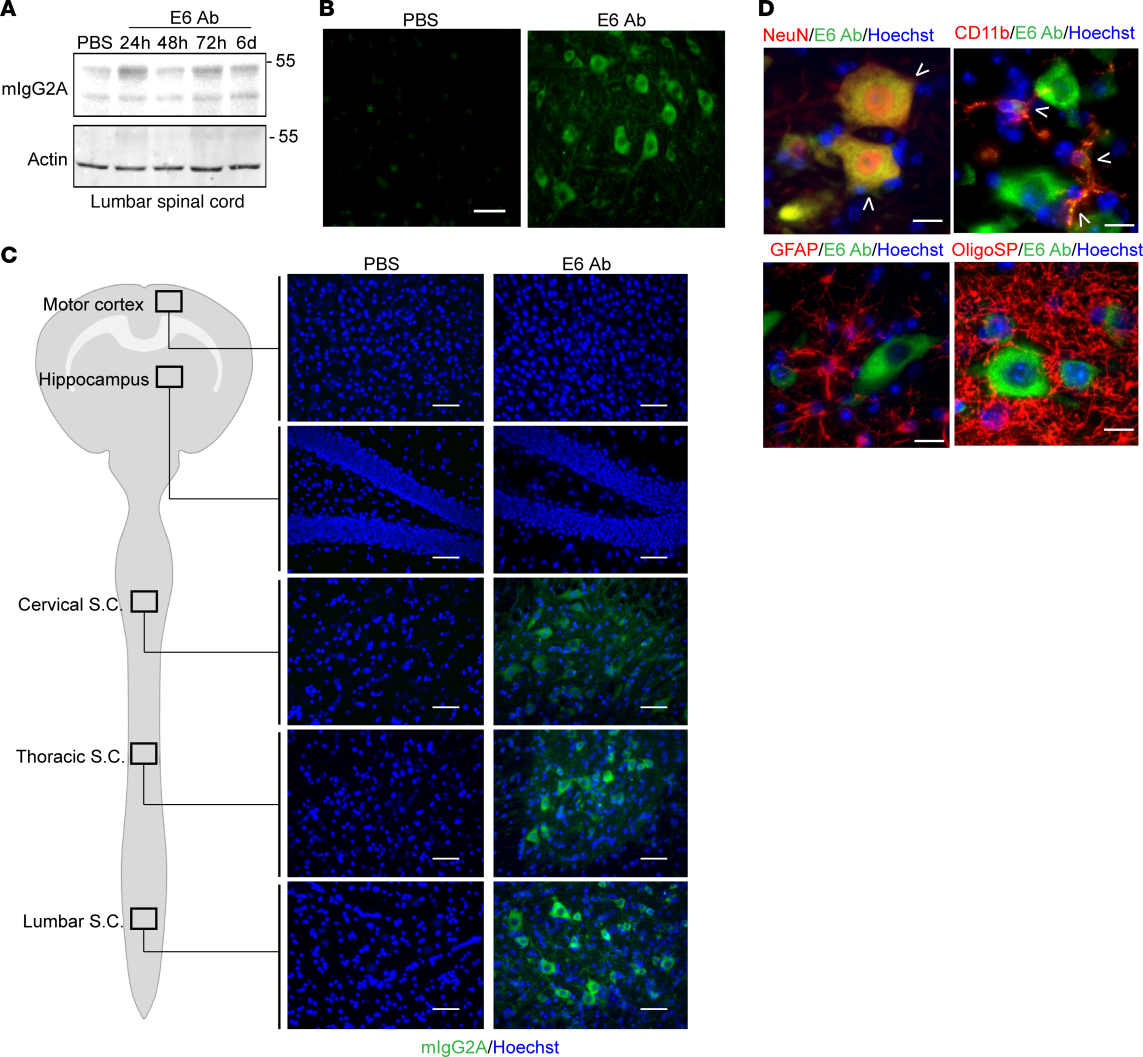
Tissue and cellular distribution after intrathecal delivery. (**A**) Western blot for mIgG2A in total lysate from lumbar spinal cord of mice injected intrathecally with E6 or equal volume of PBS. Actin was used as loading control. (**B**) E6 Ab distribution in coronal sections of lumbar spinal cord. Tissues were analyzed from mice after 24 hours from PBS treatment and 48 hours after E6 Ab treatment. Signal represents mIgG2A (green). Scale bar: 50 μm. (**C**) Representative images of the E6 antibody (mIgG2A staining, green; merged with Hoechst, blue) distribution in the lumbar, thoracic, and cervical regions of the spinal cord (S.C.) and brain (hippocampus and motor cortex) after 5 weeks of repeated intrathecal injections. Scale bar: 50 μm. The analyzed areas are highlighted in the schematic representation of the CNS. Representative pictures for control antibody are shown in Supplemental Figure 7C. (**D**) Representative channels merged images of immunofluorescence for E6 antibody (mIgG2A staining, green); markers for neurons (NeuN, red), microglial cells (CD11b, red), astrocytes (GFAP, red), and oligodendrocytes (OligoSP, red); and nuclei (Hoechst, blue) in ventral horns of lumbar spinal cord after 5 weeks of repeated injections. Arrowheads show colocalization. Scale bar: 20μm. Single channels pictures are shown in Supplemental Figure 7D. Results in **B**, **C**, and **D** were performed in 3 mice per conditions and confirmed in multiple sections from the areas of interest. The control antibody used was clone 807.33.

ALS/FTLD is a pathology that affects neurons in both the brain — in particular the frontal and temporal cortices — and the spinal cord. With a therapeutic perspective, we investigated various ways to deliver the E6 antibody in *TDP43A315T* transgenic mice with the aim of targeting neurons in the cortex and anterior horn of the spinal cord. Here, the most used ways of delivery of an antibody (31) have been tested considering the less invasive techniques — i.e., intranasal (Figure 4) and i.p. (Figure 5) — but also the most efficient in targeting the CNS — i.e., intracerebroventricular (ICV) (Figure 6) and intrathecal (Figure 7). Single injections were performed and tissues were analyzed at different time points to evaluate distribution and durability of each antibody.

We firstly evaluated intranasal delivery by treating mice with a total of 100 μg of E6 antibody or equal volume of PBS, and we then sacrificed them after 24, 48, and 72 hours (Supplemental Figure 4A). Western blot was performed on total lysates from olfactory bulb, hippocampus, frontal and posterior cortices, cerebellum, brainstem, and cervical, thoracic, and lumbar spinal cord (Figure 4A). A 50 kDa band was visible in mice treated with E6 antibody soon after 24 hours, although the higher intensity was observed after 48 hours with a general decrease after 72 hours. The antibody band was clearly detectable in the olfactory bulb, but also in the hippocampus, cortex, brainstem, and the cervical region of spinal cord. We confirmed the wide distribution of the antibody at 48 hours after intranasal injection by immunofluorescence (Figure 4B), although a deeper analysis of cellular localization showed that the antibody was mainly trapped in blood vessel structures as opposed to penetrating the tissues and localizing into cells (Supplemental Figure 4B).

I.p. delivery was assessed by treating mice with a total of 500 μg of E6 antibody or control antibody, or an equal volume of PBS; mice were then sacrificed after 2, 3, and 4 days (Supplemental Figure 5A). We confirmed the presence of the antibodies in the plasma of treated mice (Figure 5A). We also found that the antibodies could penetrate circulating peripheral blood mononuclear cells (PBMCs) (Figure 5B), which could allow the diffusion of the antibodies also in tissues. When we checked for brain penetration, we were able to detect the antibodies (both E6 and CTR), after only 3 days from injection, as a strong and diffuse signal at the level of the third ventricle and a faint and occasional penetration into some neurons (Figure 5C and Supplemental Figure 5B).

ICV delivery was performed by injecting mice unilaterally into the lateral ventricle with a total of 20 μg of E6 antibody or control antibody, or an equal volume of PBS; mice were then sacrificed after 2, 4, 6, 24, or 48 hours (Supplemental Figure 6A). We confirmed the presence on the antibodies in the CSF of treated mice (Figure 6A). We then performed immunofluorescence on brain tissues of treated mice (Figure 6B and Supplemental Figure 6B), observing the presence of E6 and CTR antibodies in the ipsilateral region of the brain in the early time points after the injection. The antibodies were clearly visible, both in the cortex and in the hippocampus of treated mice, as a diffuse signal. By analyzing later time points (24 and 48 hours), we observed a neuronal uptake and internalization after 24 hours from injection in both regions analyzed. The fluorescence signal was detectable in both ipsilateral and controlateral regions. At 48 hours, there was a loss of fluorescence signal.

We finally investigated the intrathecal delivery, with the aim of targeting motor neurons, by injecting a total of 25 μg of E6 antibody or equal volume of PBS and sacrificing mice after 24 hours, 48 hours, 72 hours, or 6 days (Supplemental Figure 7A). We confirmed the presence of the antibody by Western blot in the lumbar spinal cord of mice (Figure 7A), observing a clear signal even after 72 hours from injection and a weaker presence also after 6 days. At 72 hours, immunofluorescence microscopy revealed the E6 antibody to be internalized into spinal motor neuronal-like cells (Figure 7B).

### E6 antibody can spread thorough the spinal cord after repeated intrathecal injections.

As a proof of principle, we decided to assess the in vivo potential of the full-length anti–RRM1 TDP43 antibody to rescue ALS/FTLD pathological events in the *TDP43A315T* mouse model. As these mice exhibit motor neuron disease with aging (24), we decided to carry on the therapeutic approach with the intrathecal delivery. Due to the small volumes that can be injected in the limited intrathecal space and the possibility to deliver a fresh preparation of antibody frequently, repeated intrathecal injections — rather than a continuous diffusion — was preferred. By keeping the 72 hours as the reference time, we injected 9-month-old *TDP43A315T* mice with PBS or 25 μg of E6 antibody or control antibody (*n* = 10 per group) twice a week for 5 weeks, allowing mice to receive 10 injections and a total of 250 μg (Supplemental Figure 7B).

We evaluated the diffusion of the antibodies after 5 weeks of repeated injections. The entire CNS was analyzed by immunofluorescence for the presence of mouse IgG2A (Figure 7C and Supplemental Figure 7C). Interestingly, we found that both E6 and control antibodies were able to penetrate the CNS tissues from the intrathecal space and to diffuse from the sacral portion throughout all the length of the spinal cord, targeting cells in the lumbar, thoracic, and cervical portions. Although a weak signal was also detectable in the brainstem nuclei (data not shown), no signal was detected in the cerebellum and the brain of the mice.

A careful analysis by immunofluorescence revealed that the E6 antibody was mainly taken up by neurons and microglial cells, whereas no mIgG2A signal was observed within astrocytes or oligodendrocytes (Figure 7D and Supplemental Figure 7D).

### Repeated injections of the E6 antibody reduced cytoplasmic TDP43 and nuclear NF-κB in motor neurons.

The therapeutic effect of the repeated intrathecal delivery of the E6 antibody was evaluated in *TDP43A315T* mice intrathecally injected for 5 weeks (Supplemental Figure 7B). No adverse effect, neurological symptom, weight loss, or premature death occurred in mice throughout the course of the study, strengthening the absence of toxicity of the E6 antibody previously observed in vitro.

**Figure 8:**
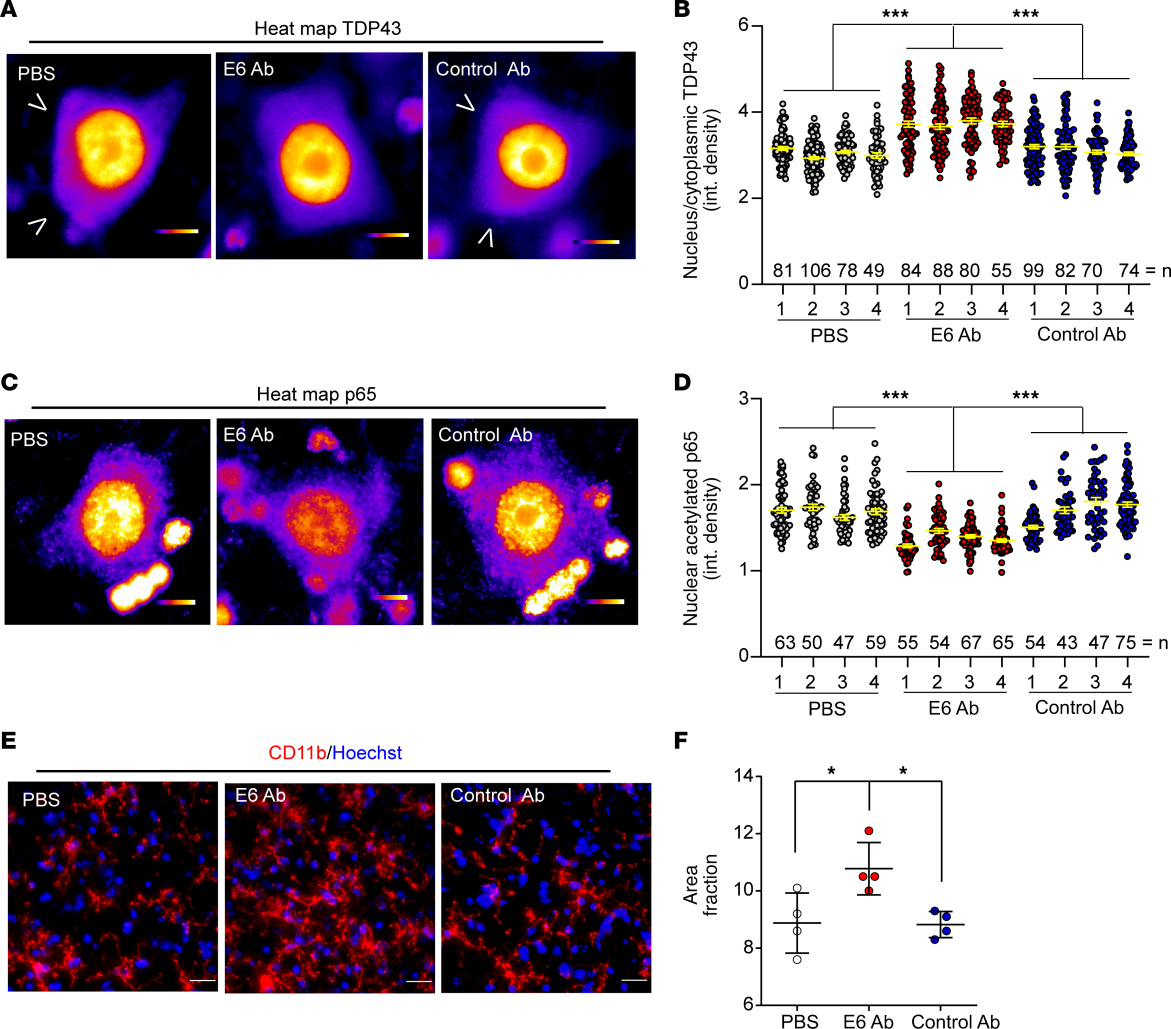
Treatment with E6 antibody reduces cytoplasmic TDP43 and nuclear p65 in lumbar motor neurons. (**A**) Representative high-magnification colorimetric heatmap images of TDP43 immunofluorescence. Single channels images are shown in Supplemental Figure 8C. Scale bar: 10 μm. Arrowheads show cytoplasmic TDP43. (**B**) Graph represents quantification of nuclear to cytoplasmic integrated density of TDP43 signal in single large neurons counted in ventral horns of mice lumbar spinal cord. Data are represented as mean ± SEM; number of counted neurons (dots) from 4 independent mice (numbered 1–4) is shown in the graph; 1-way ANOVA (F_11,934_ = 36.98, *P* < 0.0001), ***P < 0.0001 by Tukey’s multiple comparison test. (**C**) Representative high-magnification colorimetric heatmap images of p65 immunofluorescence. Single channels images are shown in Supplemental Figure 9A. Scale bar: 10 μm. (**D**) Graph represents quantification of nuclear integrated density of p65 signal in single neurons counted in anterior horn of the lumbar spinal cord. Data are represented as mean ± SEM; number of counted neurons (dots) from 4 independent mice (numbered 1–4) is shown in the graph; 1-way ANOVA (F_11,667_ = 36.39, *P* = 0.0011), ***P < 0.001 by Tukey’s multiple comparison test. (**E**) Representative images of immunofluorescence for microglial cells (CD11b, red) merged with nuclei (Hoechst, blue) performed on lumbar spinal cord of treated mice. Scale bar: 20 μm. (**F**) Graphs represent quantification of percentage of area covered by CD11b signal (area fraction). Data are represented as mean ± SEM; *n* = 4 independent mice, dots. One-way ANOVA (F_2,9_ = 6.906, P = 0.0151),*P < 0.05 by Tukey’s multiple comparison test. The control antibody used was clone 807.33.

A detailed analysis at the tissue and cellular levels was then performed on treated mice. We first evaluated the cytoplasmic levels of TDP43 in lumbar spinal cord by Western blot (Supplemental Figure 8, A and B), observing a slight but not significant reduction of cytoplasmic TDP43 in E6-treated mice. Since we observed a neuronal-specific uptake of the antibody, the analysis of TDP43 mislocalization was then performed by immunofluorescence, focusing in particular on large neurons present in the ventral horns of the lumbar spinal cord (Figure 8A and Supplemental Figure 8C). We analyzed 4 mice per group (Supplemental Figure 8D) with an average of 300 large neurons (area > 250 μm^2^) counted per experimental group (Figure 8B). In these cells, we quantified the nuclear/cytoplasmic ratio of TDP43 staining, observing a significant reduction of cytoplasmic TDP43 mediated by E6 antibody.

No motor neuron toxicity was observed in treated mice as the number of ChaT^+^ cells (area > 250 μm^2^) per section remained unaltered in the 3 different experimental groups (Supplemental Figure 2, E and F).

Another detrimental effect of TDP43 proteinopathy considered in this study is the hyperactivation of NF-κB. We previously demonstrated that the E6 antibody was able to reduce NF-κB in vitro; therefore, we decided to verify this ability in vivo after repeated intrathecal injections.

We firstly analyzed large neurons of the lumbar spinal cord ventral horns (Figure 8C and Supplemental Figure 9A), since it has been demonstrated that NF-κB activation in neuronal cells contributes to cell sensitivity to toxic pathways (32). We analyzed 4 mice per group (Supplemental Figure 9B), with an average or 200 large neurons (area > 250 μm^2^) counted per experimental group (Figure 8D). In these cells, we measured the nuclear signal of acetylated p65, a known form of activated p65 (33, 34), finding a significant reduction of nuclear p65 mediated by E6 antibody treatment. Interestingly, we observed a correlation between the presence of the E6 antibody and the reduced nuclear levels of p65 in neurons (Supplemental Figure 9C).

We then analyzed the effect of the E6 antibody on NF-κB modulation in glial cells by quantifying microglial (CD11b staining) and astrocyte (GFAP staining) reactivity in lumbar spinal cord of treated mice. Although the antibody was able to localize in microglial cells and to reduce microglial NF-κB activity in vitro, we did not observe any reduction of microgliosis after E6 treatment (Figure 8, E and F). On the contrary, the treatment with the E6 antibody induced a general microglial activation. No significant changes were observed, in terms of astrogliosis (Supplemental Figure 9, D and E).

## Discussion

Here, we report for the first time to our knowledge a mouse monoclonal full-length antibody (named E6) generated against the RRM1 domain of TDP43 that specifically recognized cytoplasmic TDP43 species in cellular systems in mouse models and in human samples. Moreover, we show that repeated intrathecal injections of the E6 antibody resulted in large neuron penetration of the antibody and mitigation of cytoplasmic TDP43 mislocalization. Finally, evidence is presented that E6 antibody–mediated TDP43 degradation may involve the TRIM21/proteasome and lysosome degradation pathways.

Over the past few decades, increasing efforts have been put in the use of therapeutic antibodies for neurodegenerative disorders, such as AD or PD (14). In the field of ALS, immunotherapy has been firstly developed against the protein SOD1 with encouraging results (15–21). Other proteins have also been the target of immunotherapy like NogoA (35, 36), IL-10 (37), and RAN proteins for C9ORF72 repeat expansions (29, 38). Recently, we demonstrated that intracellular TDP43 is a therapeutic target for antibody-based approaches. Therein, we showed that viral delivery of a single-chain antibody against the RRM1 domain of TDP43 was able to recognize its target and to reduce the levels of cytoplasmic TDP43 accumulation, together with improvement of cognitive and motor functions in 2 mouse models of ALS/FTLD (22).

Despite these promising results, for human clinical trial, the administration of full-length antibodies would likely be safer and easier to implement than AAV-mediated transfer of single-chain antibodies. Indeed, in case of adverse effects, the direct administration of a monoclonal antibody allows modulation of treatment. Moreover, the presence of the constant fragment (Fc) in the full-length antibody gives therapeutic advantages related to cellular uptake in neurons expressing Fc receptors (39) and target degradation (30, 40). Therefore, we examined the ability of the E6 full-length antibody to mitigate TDP43 proteinopathy in the ALS/FTLD mouse model.

From previous studies, we knew that this antibody was able to recognize TDP43 in nuclear lysates of murine cells, as do commercial antibodies. Here, we confirmed the ability of the E6 antibody to bind to TDP43, but we also describe a lower affinity for the target compared with commercial mouse anti–human TDP43 N-Term (Abnova), commonly used for diagnosis or research purposes (Figure 1B). TDP43 can freely shuttle between the nucleus and cytoplasm, and in both compartments, it has important biological functions (41). When it consistently mislocalizes in the cytoplasm of cells and aggregates, TDP43 acquires pathological features, mainly losing the physiological ability to move between the nucleus and cytoplasm and to freely bind proteins or RNA (42). Here, we show that, in cellular systems, animal models, and human tissues, the E6 monoclonal antibody mainly recognizes the cytoplasmic TDP43. Interestingly, by Western blot on total cell lysates, the E6 antibody showed a preference for the 35 KDa form of the protein (Supplemental Figure 1B), which is known to be one of the pathological and cytoplasmic fractions of the protein (23). This preference for the cytoplasmic protein was clearly visible in pathological conditions where TDP43 was overexpressed and mislocalized in culture cells (Figure 1C) or in cortical and large neurons of the lumbar spinal cord of mutant TDP43–overexpressing mice (Figure 1D). In contrast to the E6 antibody, commercial antibodies against TDP43 predominantly detect the nuclear TDP43 species. It is noteworthy that the E6 monoclonal antibody recognized cytoplasmic TDP43 also in human cortical neurons of control and ALS/FTLD patients (Figure 2A) and in large neurons of the spinal cord ventral horns (Figure 2B). The E6 immunostaining of punctate structures in the cytoplasm of pathological neurons must reflect the ability of the antibody to detect aggregated TDP43. Interestingly, in physiological conditions like untransfected Hek cells (Figure 1C), nontransgenic mice (Supplemental Figure 1D), or healthy individuals (Figure 2A) — where the C-Term antibody shows mainly a nuclear TDP43 signal— E6 can recognize the physiological cytoplasmic localized protein. The lack of E6 immunostaining for TDP43 in the nucleus may reflect the masking of the epitope in the RRM1 domain, which interacts in this compartment with nucleic acids (43). The specificity of the E6 antibody for the cytoplasmic TDP43 makes it a unique antibody with therapeutic potential to target TDP43 pathology.

We demonstrate that the E6 monoclonal antibody was also able to penetrate neurons and microglial cells both in vitro and in vivo (Figure 3 and Figure 7D) and to mediate a therapeutic effect once inside cells. It has been previously described that antibodies against Tau are primarily taken up by neurons via clathrin-dependent Fcγ receptor endocytosis (30, 39). Similarly, we observed a quick uptake of the E6 monoclonal antibody by cultured neuronal and microglial cells, which appeared higher for E6 monoclonal antibody than for control. As suggested also in the case of Tau antibodies, the presence of the pathological target inside the cell may influence antibody internalization and/or retention in neurons (39). Once inside neurons, both in vitro and in vivo, the E6 antibody showed no toxicity. On the contrary, we observed a reduction in levels of cytoplasmic TDP43 (Figure 3E and Figure 8, A and B). The reduction of the pathological form of TDP43 can be explained by 2 known mechanisms of action of antibodies, both of them dependent on the Fc domain of an antibody (i.e., TRIM21-dependent proteasome degradation and the lysosome system). It has been demonstrated that, once inside the cells and bound to its target via the antigen binding fragment (Fab), an antibody can be recognized by TRIM21, an intracellular Fc receptor (44). TRIM21 is a E3 ubiquitin ligase that can mediate the coupling of the complex antibody/antigen to the proteosomal machinery, inducing a fast degradation of endogenous cytoplasmic proteins recognized by antibodies (27, 28). The TRIM21-dependent degradation mechanism has been previously demonstrated with antibodies against Tau (40) and very recently also for antibodies targeting RAN proteins (29). In this study, we demonstrate that cytoplasmic TDP43 can be degraded by the E6 monoclonal antibody and that this event was TRIM21 dependent, since inhibition of proteasome (Figure 3E), the final step in this mechanism, or overexpression of TRIM21 (Figure 3G) altered TDP43 degradation only in the presence of the E6 monoclonal antibody. Interestingly, both the E6-derived single-chain antibody previously tested (22) and the E6 full-length form can mediate a reduction of the cytoplasmic TDP43 in large neurons of about 30% compared with control antibodies. The 2 forms of the E6 antibody, which share the same antigen binding domain with strong specificity for the pathological protein, were demonstrated to overlap in the final effect of degrading TDP43, although their mechanisms of action are different. The single-chain antibody indeed acts as a flag on TDP43 for cells that enhance the ubiquitination of the protein for the proteasome and the autophagic pathways. Instead, the full-length antibody, which possess the Fc domain, once inside the cell is recognized by a strong and effective machinery of degradation represented by TRIM21 and the proteasome. Moreover — as it happens for anti-Tau antibodies, reported to induce Tau degradation also by the endosome/autophagosome/lysosome system (30) — here, we demonstrate that a small portion of E6 colocalized with both late endosomes (Figure 3H) and lysosomes (Figure 3I), combining to the demonstrated TRIM21-mediated pathway, and we demonstrate the possibility that the cytoplasmic TDP43, bound to E6, might be degraded also via the lysosomes machinery.

Another beneficial effect of the E6 antibody was the reduction of NF-κB activation in large neurons of the lumbar spinal cord (Figure 8, C and D). This phenomenon may be due to either a reduction of the cytoplasmic TDP43 or to the antibody block of the TDP43/p65 interaction (22). As demonstrated previously (32), a reduced NF-κB activation in neuronal cells protects them from detrimental toxic stimuli.

Macrophages and microglial cells express Fcγ receptors as neurons (45, 46), supporting the presence of the E6 monoclonal antibody in circulating PBMC and microglial cells both in vitro and in vivo (Supplemental Figure 3B, Figure 5B, and Figure 7D). In vitro, we were able to demonstrate a reduction of NF-κB activation mediated by the antibody (Figure 3C). In contrast to the in vitro results, we registered an activated phenotype for microglial cells after E6 antibody treatment in mice (Figure 8, E and F). This might reflect a protective phagocytic phenotype, which can help in neuronal TDP43 clearance (47) and can also facilitate the uptake of the antibody, or an immunogenic effect of the antibody on microglial cells induced by the binding between the Fc fragment and the Fc receptors present in these cells (48, 49). In this regard, the previously studied E6-derived single-chain antibody, which was lacking the Fc portion, did not induce any immunogenicity but instead reduced NF-κB activity, confirming the ability of the Fab in blocking the TDP43/p65 interaction and in reducing inflammation. The discrepancy between the results obtained in vitro and in vivo with the full-length E6 antibody might also be due to the amount of antibody delivered and the duration of the treatment (2.5 μg/mL for 6 hours in cells and a total of 250 μg over a period of 5 weeks for mice). We speculate that a longer treatment with a lower amount of antibody delivered or less frequent injections could result in a reduced microgliosis also in vivo. This, together with the efficiency of the E6 antibody in mediating the degradation of the cytoplasmic TDP43, might eventually result in a stronger therapeutic effect in ALS/FTLD mouse models.

Finally, few considerations should be given for the anti-TDP43 antibody delivery methods described in this study. Here, we show for the first time to our knowledge a comprehensive description of CNS distribution in TDP43 mutant mice of a mouse IgG2A antibody against TDP43 after systemic or local administration commonly used for antibody delivery (31). We first analyzed 2 main noninvasive delivery methods ( i.e., the i.p. and the intranasal methods). I.p. injection (Figure 5) unfortunately yielded only a small and weak distribution of the antibody in the brain, mainly limited to the ventricular area. Although not suitable for CNS delivery, we demonstrated a sustained and long-lasting presence of the antibody in the bloodstream and in blood cells, which might be therapeutically useful in case of TDP43-induced peripheral pathological events like, for example, gastrointestinal problems described in mutant TDP43–overexpressing mice (50–52). On the same side, the internalized E6 antibody in peripheral blood cells might target and modulate TDP43 mislocalization (53) and the decreased survival ability of PBMC documented for ALS patients (54, 55), or it could help the antibody to reach the CNS through infiltrating lymphocytes (56, 57).

Interesting results were obtained for intranasal delivery (Figure 4). Here, we confirmed that intranasal administration of an antibody yielded a strong and wide distribution throughout the entire brain (58), which was maintained even after 3 days from delivery. We also demonstrated that intranasal delivery allowed the antibody to reach the brainstem and the cervical tract of the spinal cord. Unfortunately, as already reported (58), the single intranasal administration failed to permit a cellular penetration of the antibody, which remained mainly localized in blood vessels. Perhaps repeated intranasal administration may lead to a better cellular localization, but this hypothesis merits further investigation.

We also analyzed 2 main ways of delivery into the CSF — i.e., the ICV (Figure 6) and the intrathecal (Figure 7) methods — which, although more invasive than i.p. and intranasal, have the great advantage of bypassing the major obstacle for macromolecules delivery, the blood-brain barrier (BBB) (13). At 2 hours after ICV injection, we were able to detect the antibody spread from the lateral ventricle to cortical and hippocampal regions of the ipsilateral hemisphere. With time, the antibody disseminated to the contralateral hemisphere. At 24 hours after single injection, there was evidence of massive penetration of antibodies into cells. At 48 hours, the signal was then reduced in tissue but still present in the CSF.

Efficient results in terms of distribution and cellular penetration were finally obtained with intrathecal delivery. We demonstrated the presence of the antibody in the spinal chord up to 3 days after a single intrathecal delivery, which yielded an appreciable penetration in large neurons of the lumbar spinal cord. We demonstrated that a treatment with repeated intrathecal injections for 5 weeks was safe and feasible for transgenic 9-month-old mice expressing mutant TDP43 and with evidence of target recognition (Figure 7, C and D). Indeed, by exploiting the CSF flux, the antibody diffused throughout the entire length of the spinal cord until the cervical region and penetrated in ALS pathological cells (e.g., motor neurons). The fact that neurons possess the Fc receptors and a higher level of the pathological target makes this cellular population a good target for the antibody that, once internalized, mediates the therapeutic effect previously discussed. It might be interesting in the future to administer the antibody through intrathecal cervical injection. This technique, used for example for the administration on analgesic compounds in pain treatment, might allow our antibody to achieve a better cervical spinal cord and brain diffusion.

In conclusion, we demonstrate that a full-length monoclonal antibody against the TDP43-RRM1 domain, named E6, (a) recognized specifically cytoplasmic TDP43 in cellular, animal, and human samples; (b) mediated cytoplasmic TDP43 degradation in large neurons of the spinal cord, likely through TRIM21 and lysosome-dependent mechanisms; and (c) reduced NF-κB activation. Moreover, we describe and compare 4 ways of antibody delivery to the CNS that highlighted (d) the therapeutic validity of repeated intrathecal injections for ALS in targeting large neurons throughout the entire length of the spinal cord ventral horns, (e) the capability of continuous ICV delivery to target cortical and hippocampal neurons, and (f) the potential of a wide brain distribution after intranasal delivery. These results demonstrate the feasibility of a full-length antibody-based therapeutic intervention against cytoplasmic TDP43 in the context of ALS/FTLD.

## Methods

Methods related to cell cultures transfection and test, ELISA array, IHC and immunofluorescence, protein extractions, dot blots, and Western blots can be found in Supplementary Methods. Full and uncut gels from Western and dot blot experiments are available as online Supplemental Material.

### Aim and experimental design of the study.

In this study, we aimed to (a) test the target specificity, (b) the in vivo distribution, and (c) the therapeutic efficacy of the E6 full-length monoclonal antibody. We analyzed the antibody specificity in TDP43-overexpressing neuronal cells, animal tissues, and ALS/FTLD human samples. The in vivo distribution was analyzed at different time points in *TDP43A315T* mice after single i.p., intranasal, ICV, or intrathecal injections. The therapeutic efficacy was analyzed in microglial and neuronal cell lines, as well as in *TDP43A315T* mice that received repeated intrathecal injections twice a week for 5 weeks.

Experiment implementation and data collection were performed in a blinded manner for all animal studies (operators were kept blind when administering treatment or analyzing tissues). Data collection was performed in a blinded manner for in vitro experiments (operators were kept blind on treatments). To evaluate the therapeutic efficacy in mice, treatment randomization was applied, although sex balance was taken in consideration among groups. No data were excluded from analyses.

### Anti-TDP43 and control full-length monoclonal antibodies.

Both anti-TDP43-RRM1 domain (E6) monoclonal antibody (mIgG2A), produced as previously described (22), and the control (807.33) mouse monoclonal antibody (mIgG2A) anti-G1 protein of La Crosse virus (ATCC, CRL-2290) were purchased from Medimabs after purification and lyophilization of the antibody from media of hybridoma cells (Supplemental Table 1). For direct fluorescence detection, E6 and 807.33 antibodies were conjugated with Alexa Fluorochrome (807.33-488 and E6-488) using an Alexa Fluor 488 Antibody Labelling Kit (Thermo Fisher Scientific) following manufacturer’s instructions and 6-hour dialysis against PBS to remove unbound die and sodium bicarbonate.

### Patients.

Control samples were obtained from a 70-year-old man with no history of neurodegenerative disorder who died from multiple organ failure (identifier 18N00032). Experimental samples were obtained from a 60-year-old man affected with TDP43-related FTLD (identifier 15N00204) and from a 54-year-old man affected with sporadic ALS (identifier 20N00264). Identifiers refer to the Neurodegenerative Diseases Brain Bank of Angers University Hospital.

### Mice treatments and tissues collection.

*TDP43A315T* mice (24) were identified by PCR on DNA form ear biopsies and constantly maintained, after more than 20 backcrosses, on a C57BL/6 strain (Charles River Laboratories) as a colony at the CERVO Brain Research Centre animal facility under standard conditions. Antibody distribution analyses were performed on female *TDP43A315T* mice (*n* = 31; average age, 10 months), whereas the antibody therapeutic efficacy was assessed in 9-month-old *TDP43A315T* mice (*n* = 30 in total; *n* = 7 males and *n* = 3 females per group). Nontransgenic mice (*n* = 3) from the same background and age (littermates) of *TDP43A315T* mice were used for immunofluorescence experiments.

For i.p. administrations, mice were injected with 500 μL of 0.9% saline or 1 mg/mL purified monoclonal antibody. For any other antibody delivery, mice were anesthetized with 2% isoflurane during all the procedures and were posttreated with s.c. buprenorphine (0.05 mg/kg) to prevent postoperative pain, except for intranasal delivery. For intranasal administration, anesthetized mice were placed on their backs, and droplets of 0.9% saline or 1 mg/mL purified monoclonal antibody solution were dropped into the mouse nostrils using a pipette. A total of 50 μL of solution per mouse (25 μL per nostril) was administered. For ICV delivery, mice were placed in a stereotaxic apparatus (David Kopf Instruments), and the right ventricle was then reached with a 33-gauge stainless steel cannula (Plastics One) that was connected to a 25 μL Hamilton syringe with an intramedic polyethylene tubing (PE-50; Clay Adams). A total of 0.9% saline, or 2 mg/mL purified monoclonal antibody solution, was administered in a maximum volume of 4 μL over 4 minutes by means of a microinjection pump (model A-99; Razel Scientific Instruments). Intrathecal injections were performed as previously described (18). Briefly, a total of 20 μL volume of PBS or 1.25 mg/mL purified monoclonal antibody solution was slowly injected into the dura (L4–L5 intervertebral space) using a sterile Hamilton syringe. The presence of a reflex contraction of the hind legs or the tail was considered indicative of a successful administration. The syringe was removed 1 minute after the end of the injection to minimize CSF and solution leakage. Tissues were collected after deep anesthesia with 10 μL/g of pentobarbital (12 mg/mL, CERVO Brain Research Centre animal facility). For protein analyses, mice were perfused intracardially with cold 0.1M phosphate buffer. Tissues were rapidly collected, frozen on dry ice, and stored at –80°C until used for protein extraction. For histological analyses, mice were first perfused intracardially with 0.1M cold phosphate buffer and were subsequently perfused with cold 4% paraformaldehyde (PFA). Tissues were then collected, postfixed for 24 hours in 4% PFA, and cryoprotected in sucrose 30%.

CSF was collected as previously described (59) from the cisterna magna of mice, centrifuged at 13500*g* for 5 minutes at 4°C, and stored at –80°C until analysis.

Plasma and PBMC from mice were obtained as previously described (60, 61) with a few modifications. Briefly, mice were deeply anesthetized, and blood was sampled by intracardiac drawing and transferred into EDTA containing vials (Sarstedt). Blood was then diluted 1:1 with PBS and stratified on 1 volume of Ficol Plaque Premium 1.084 density (GE Healthcare). After centrifugation at 400*g* for 40 minutes, plasma was collected and stored at –80°C until use for analysis, whereas the buffy coat, containing PBMC, was transferred into a new tube, resuspended with DPBS (Thermo Fisher Scientific), and centrifuged first at 500*g* for 15 minutes and after at 400*g* for 10 minutes. Pellet containing PBMC was stored at –80°C until use for analysis. All centrifugation steps were done at room temperature.

### Image acquisition and analysis.

Slides with stained human frontal cortex samples were placed on the stage of an inverted motorized microscope NIKON ECLIPSE Ti-E (Nikon Instruments Europe) equipped with a CFI SR APO TIRF 100× ON1.49 objective, a Perfect Focus System, and a Total Internal Reflection Fluorescence (TIRF) ILas2 module (Roper Scientific). Acquisition of images was obtained using Metamorph 7.7 software (Molecular Devices). Image sequences were acquired with a single photon–sensitive camera Evolve 128TM EMCCD 512 × 512 imaging array, 16 × 16 μm pixels (Photometrics).

For low-magnification fluorescence imaging, slides with stained cells or animal tissues were placed on the stage of a DM5000 B wide-field microscope (Leica Microsystems) connected to a CTR5000 station and a Mi-150 Fiber Optic Illumination System (Dolan-Jenner) and equipped with 10×/0.30 Ph1 HC PL Fluotar, 20×/0.50 Ph2 HC PL Fluotar, and 40×/0.75 Ph2 HCX PL Fluotar objectives. Images were acquired using a Digital FireWire Monochrome Camera (DFC350 FX) controlled by Leica Application Suite software version 4.8.0. For high-magnification fluorescence imaging, slides with animal tissues were placed on the stage of a Axio Imager Z1 wide-field microscope (Zeiss) equipped with EC-plan Neofluar 40×/0.75 Ph2 M27, 63× Plan Apochromat 63×/1.40 Oil DIC M27, and EC-plan Neofluar 100×/1.30 Oil Ph3 M2 oil immersion objectives. Images were acquired using an AxioCam MRm camera controlled by AxioVision software (Carl Zeiss) version 4.8.1.0.

ImageJ software was used for image reconstruction and morphometric analysis. To assess the number of motor neurons in ventral horns, neurons stained with anti-ChaT antibody were counted on pictures acquired at low magnification (20×). The nucleus/cytoplasmic ratio of TDP43 was determined for each cell at high magnification (63×) as described before (22, 62). To quantify NF-κB activation in neurons, the mean intensity of acetylated NF-κB signal was measured in the nucleus and corrected to the mean cytoplasmic signal of the same cell (considered as basal signal). TDP43 mislocalization and nuclear acetylated p65 were analyzed only in large (area > 250 μm^2^) motor neuron–shaped cells of the ventral horns of the spinal cord with visible nucleoli. The analysis was done by an experienced neuropathologist. For astrogliosis and microgliosis assessments, an automated intensity detection threshold (IJ_IsoData) was applied to images acquired at medium magnification (40×), and elements higher than 30 μm were selected and analyzed for area distribution (22).

### Statistics.

Statistical analyses were performed using PRISM software version 5.0 for Windows (GraphPad). Unpaired 2-tailed *t* test or 1-way ANOVA followed by Tukey’s post hoc test for multiple-comparison corrections were performed according to the experimental design. Normal distribution and the homoscedasticity of data were verified using Shapiro–Wilk’s test. Differences were considered significant when *P* < 0.05. No power analysis was performed before animal treatment, but sample size is consistent with other studies in the field (63).

### Study approval.

Postmortem cryopreserved brain samples were obtained from the Neurodegenerative Diseases Brain Bank of Angers University Hospital (national identifier BB-0033-00038, Regional Ethics Committee declaration no. DC-2011-146). The Animal Care Ethics Committee of Université Laval approved all in vivo experimental protocols used in this study. Experiments were carried out in accordance with the Guide to the Care and Use of Experimental Animals of the Canadian Council on Animal Care.

## Author contributions

SP designed the study; performed ELISA, immunofluorescence, and Western blot experiments in mice and all manipulation of culture cells; analyzed the data; performed statistical analyses and data interpretation; prepared figures; and wrote the manuscript. PC contributed in study design and tissue analysis of repeated injected mice; performed immunofluorescence experiments in cells, mice, and human tissues; and contributed in preparing figures and writing the manuscript. GS performed all the injections in mice. LR helped with Western blot experiments in mice and cells. PJC helped in collecting tissues for antibody distribution analysis and in purifying PBMC. KD performed preliminary experiments on full-length antibody. CB performed PCR for mice genotyping. JPJ supervised the study and revised the manuscript. All authors agreed to be personally accountable for contributions and ensured that questions related to accuracy and integrity of any part of the work are appropriately investigated, resolved, and documented by literature. All authors read and approved the final manuscript.

## Supplementary Material

supplemental data

## Version 1 10/06/2020

In-Press Preview

## Version 2 11/05/2020

Electronic publication
