# Supplementary material for: Monoclonal full-length antibody against TAR DNA binding protein 43 reduces related proteinopathy in neurons
Source: JCI Insight. 2020 Nov 5;5(21):e140420. doi: 10.1172/jci.insight.140420 (PMC7710295; doi:10.1172/jci.insight.140420)
Supplement: supplemental data [file jciinsight-5-140420-s190.pdf]

Supplementary Material

Supplementary Figure 1

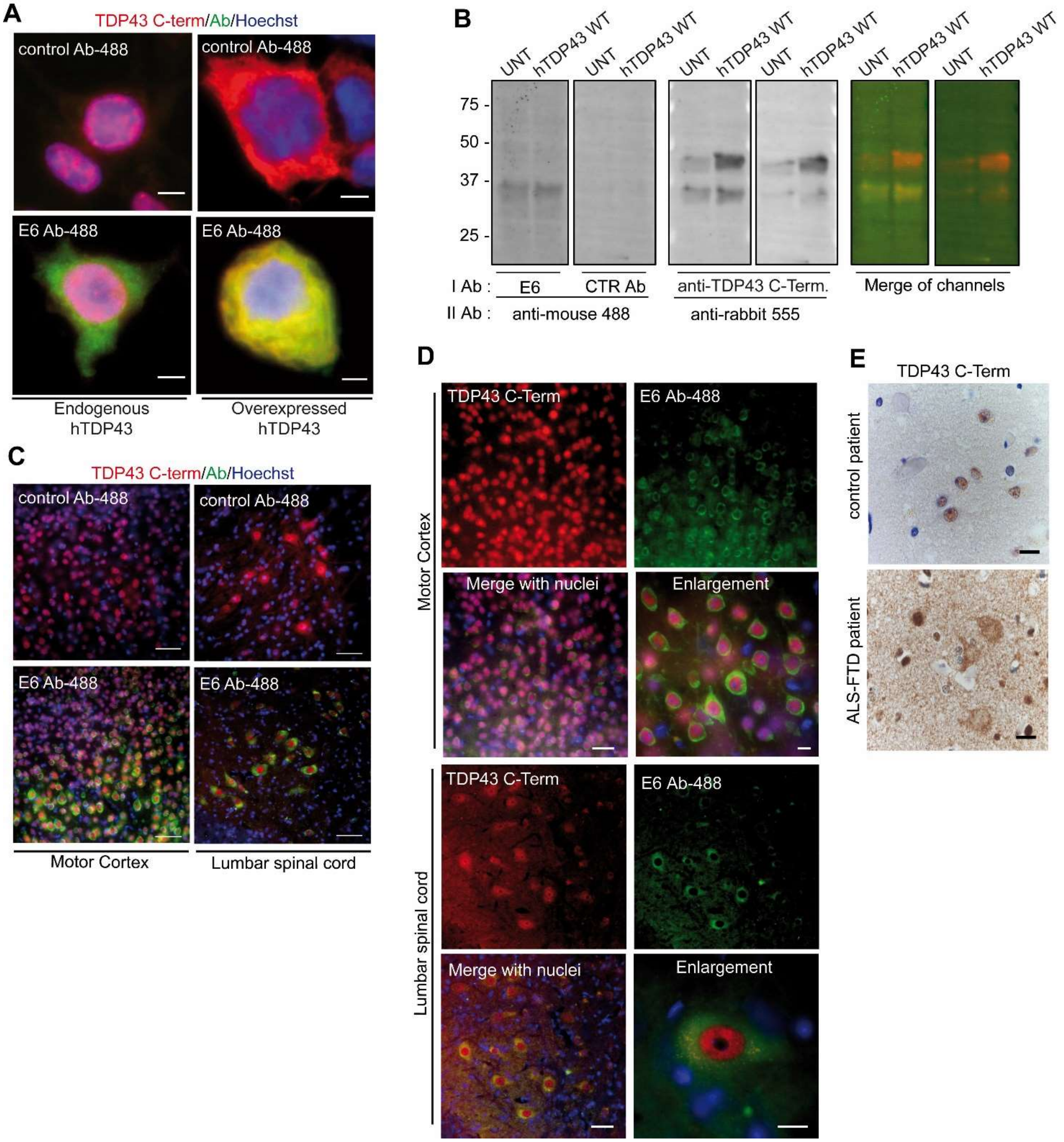

Supplementary Figure 1. E6 antibody recognizes cytoplasmic TDP43. (A) Representative merged channels immunofluorescence with anti-TDP43 C-Term (Proteintech, red), control- or E6-488 conjugated (green) and nuclei (Hoechst, blue) on fixed HEK293 cells in standard conditions or after overexpression of *hTDP43-WT*, scale bar=5 µm. Single channels images are shown in figure 1C. **(B)** Representative western blot on lysates from Hek293 cells untransfected (UNT) or transfected with *hTDP43WT*. Membranes were probed with E6 or control antibody together with anti-TDP43 C-Terminal antibody (Proteintech) and signal was visualised with anti-mouse 488 (green) and anti-rabbit 555(red). **(C)** Representative merged channels immunofluorescence with anti-TDP43 C-Term (Proteintech, red), control- or E6-488 conjugated antibodies (green) and merge with nuclei (Hoechst, blue) on motor cortex or lumbar spinal cord of 10 months old *TDP43A315T* mice, scale bar=50 µm.

Single channels images are shown in figure 1D. **(D)** Representative immunofluorescence with anti-TDP43 C-Term (Proteintech, red), control- or E6-488 conjugated antibodies (green) and merge with nuclei (Hoechst, blue) on motor cortex or lumbar spinal cord of 10 months old non-transgenic mice, scale bar=50 µm and 10 µm in enlarged (5X) pictures. **(E)** Representative immunohistochemistry performed with anti-TDP43 C-Term on prefrontal cortex of a control non-neurodegenerative patient and an ALS-FTD patient, scale bar=20 µm. Experiments in panels A and B were repeated more than three times and in C and D on three animals per conditions. Control antibody (clone 807.33).

Supplementary Figure 2

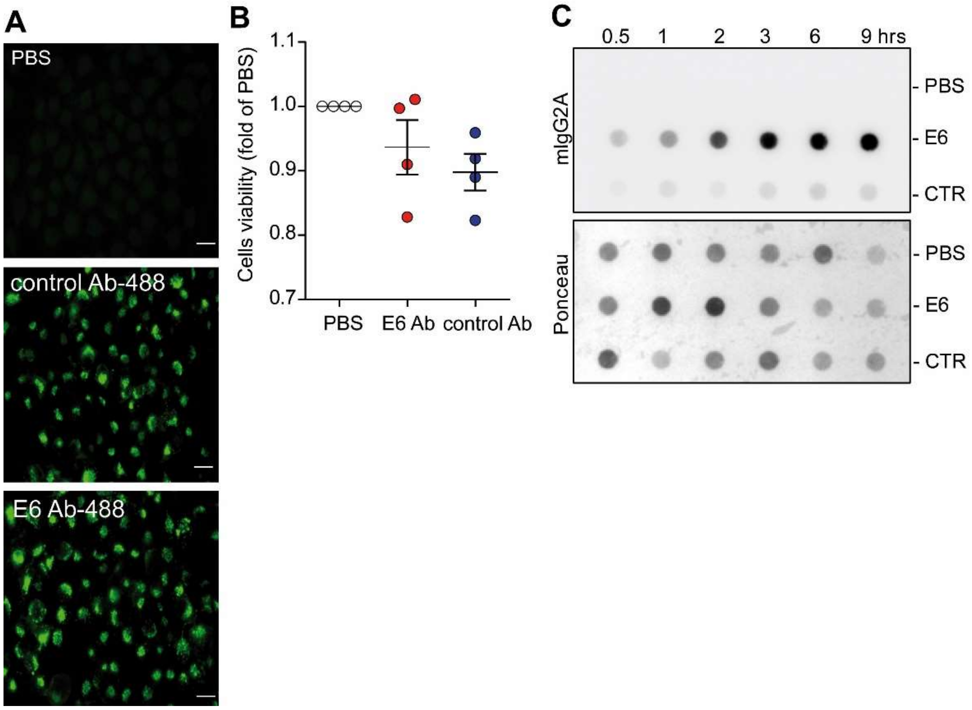

Supplementary Figure 2. E6 full-length antibody is internalized in culture cells.
(A) Representative fluorescence microscopy imaging showing control- or E6-488 conjugated antibodies (green) internalized in the cytoplasm of neuronal N2A cells after 24h treatment, scale bar=20 µm. Merge with nuclei is shown in figure 3B. Images were acquired with automatic light intensity regulation. **(B)** Viability assay on neuronal N2A cells treated 24h with 10 µg/ml E6 or control antibodies. Data are mean±SEM, n=4 independent experiments (dots) and are expressed as fold of PBS treated, One-way Anova (interaction $F_{2,9}$=3.042 P=0.0979). **(C)** Representative dot blot showing mIgG2A reactivity form control- or E6 antibodies in total lysates of neuronal *GFP-mNLS-TDP43* transfected N2A cells after CHX and 10 µg/ml E6 or control antibody treatments. Ponceau was considered as loading reference. Experiments in panels A and C were repeated more than three times. Control antibody (clone 807.33).

Supplementary Figure 3

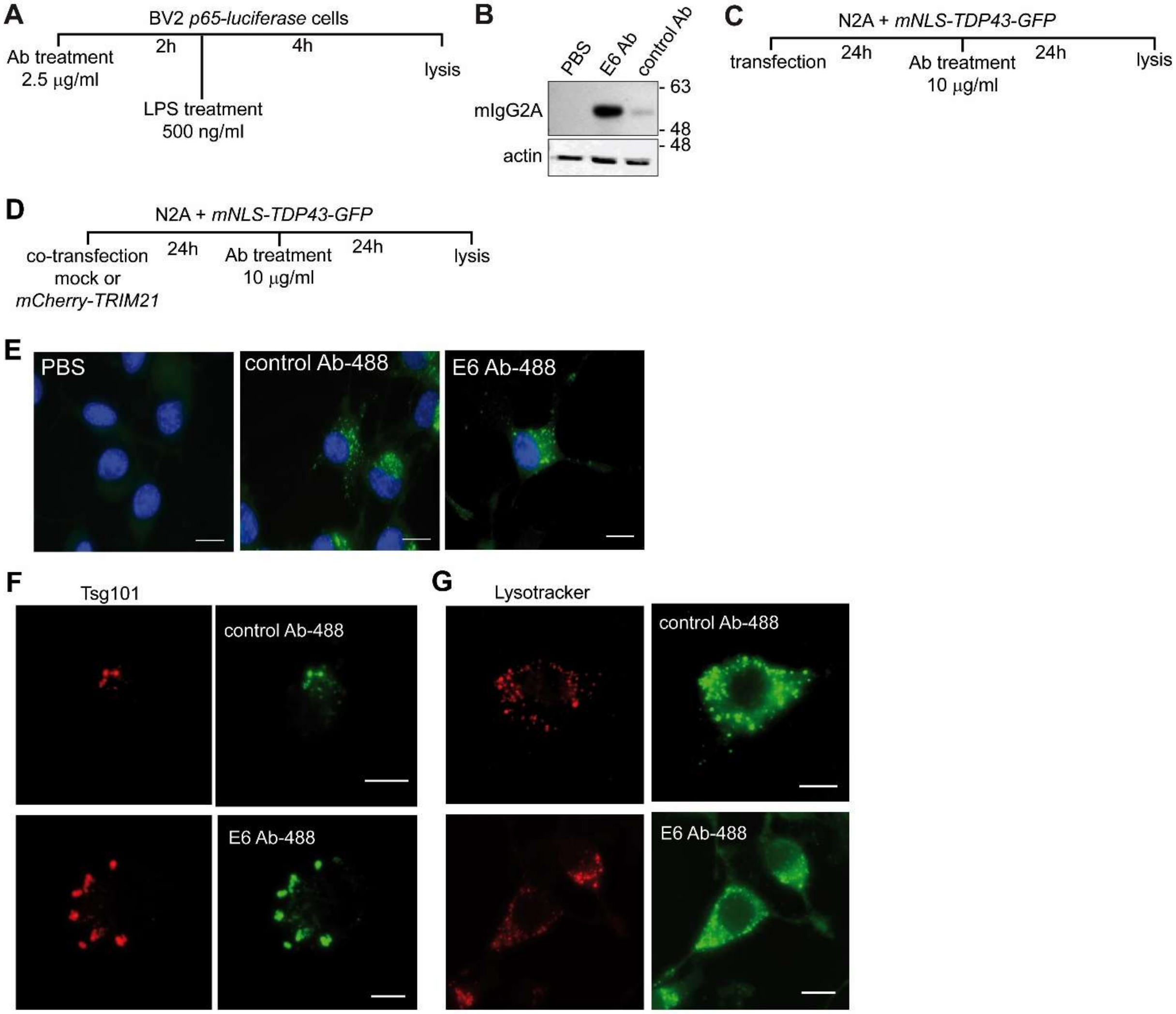

Supplementary Figure 3. E6 full-length antibody reduces cytoplasmic TDP43 and NF-KB activation in vitro. (A) Schematic representation of BV2-*p65-luc* microglial cells treatment analysed in panel B and figure 3C. (**B**) Representative western blot showing the mIgG2A antibodies in total lysates of BV2-*p65-luc* cells, actin was considered as loading reference. **(C)** Schematic representation of N2A cells transfection and treatments analysed in figure 3D, E. After 24h from transfection medium was changed and cells were treated for 24h with 10 µg/ml of antibodies and CHX. (**D**) Schematic representation of N2A cells transfection and treatments analysed in figure 3F,G. After 24h from transfection medium was changed and cells were treated with 10 µg/ml of antibodies and CHX (cycloheximide)+DMSO (dimethyl-sulfoxide) or CHX+MG-132. **(E)** Representative fluorescence microscopy imaging showing control- or E6-488 conjugated antibodies (green) internalized in the cytoplasm of neuronal N2A cells after 24h treatment, scale bar=10 µm. Images were acquired with automatic light intensity regulation. (**F**, **G**) Representative single channels fluorescence microscopy imaging for 488-conjugated antibodies (green) with Tsg101 (red) late endosomal marker (**F**) or lysotracker (red) (**G**) in neuronal N2A cells treated 24h with 10 µg/ml of antibodies, scale bar=20 µm. Images were acquired with automatic light intensity regulation. Merged channels for antibodies and Tsg101 or lysotracker signals are shown in figure 3H-I. Experiments showed in panels B, E, F and G were repeated at least three times. Control antibody (clone 807.33).

Supplementary Figure 4

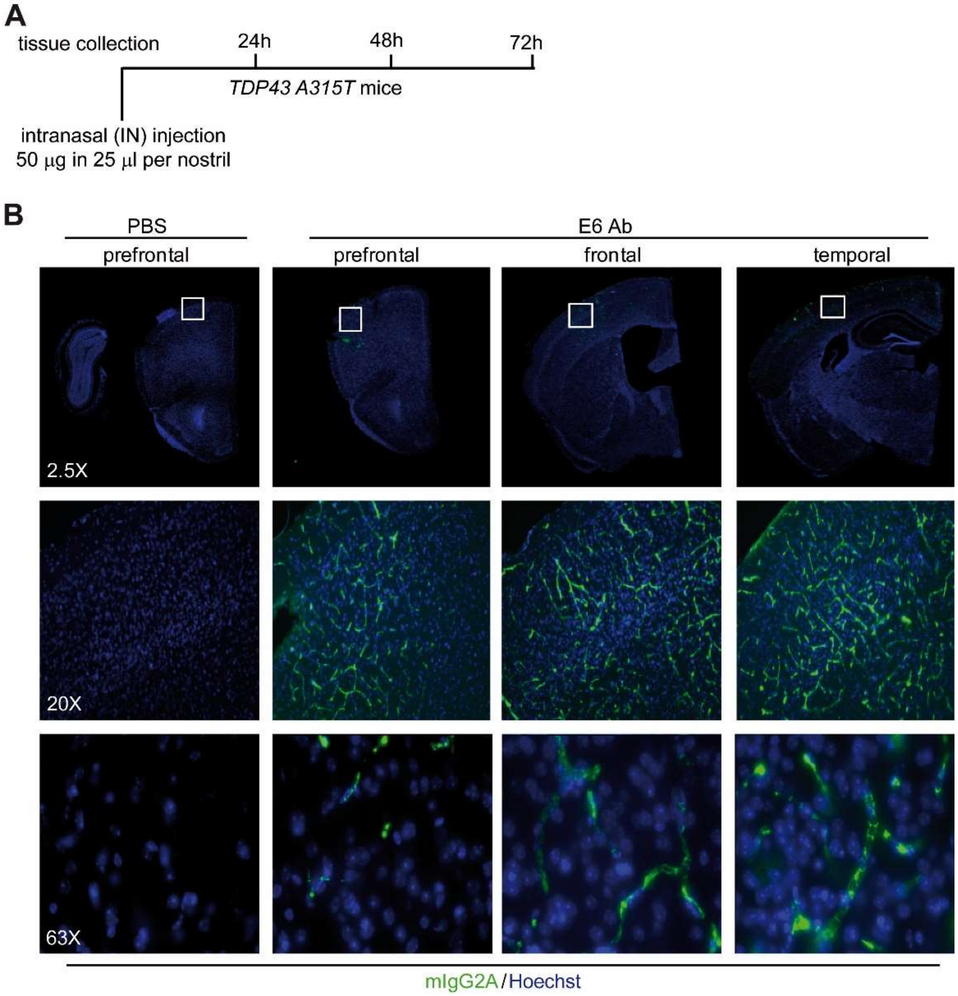

Supplementary Figure 4. Tissue and cellular distribution after intranasal (IN) delivery. (**A**) Schematic representation of intranasal delivery and time points of tissue collection. (**B**) E6 Ab distribution in coronal sections of brain. Mouse PBS treated was sacrificed after 24h whereas E6 Ab treated after 48h. Serial enlargements (2.5X, 20X, 63X) were taken from highlighted regions (square) in prefrontal, frontal and temporal cortex of mice. One mouse per condition was used but results were confirmed in multiple sections from the areas of interest.

Supplementary Figure 5

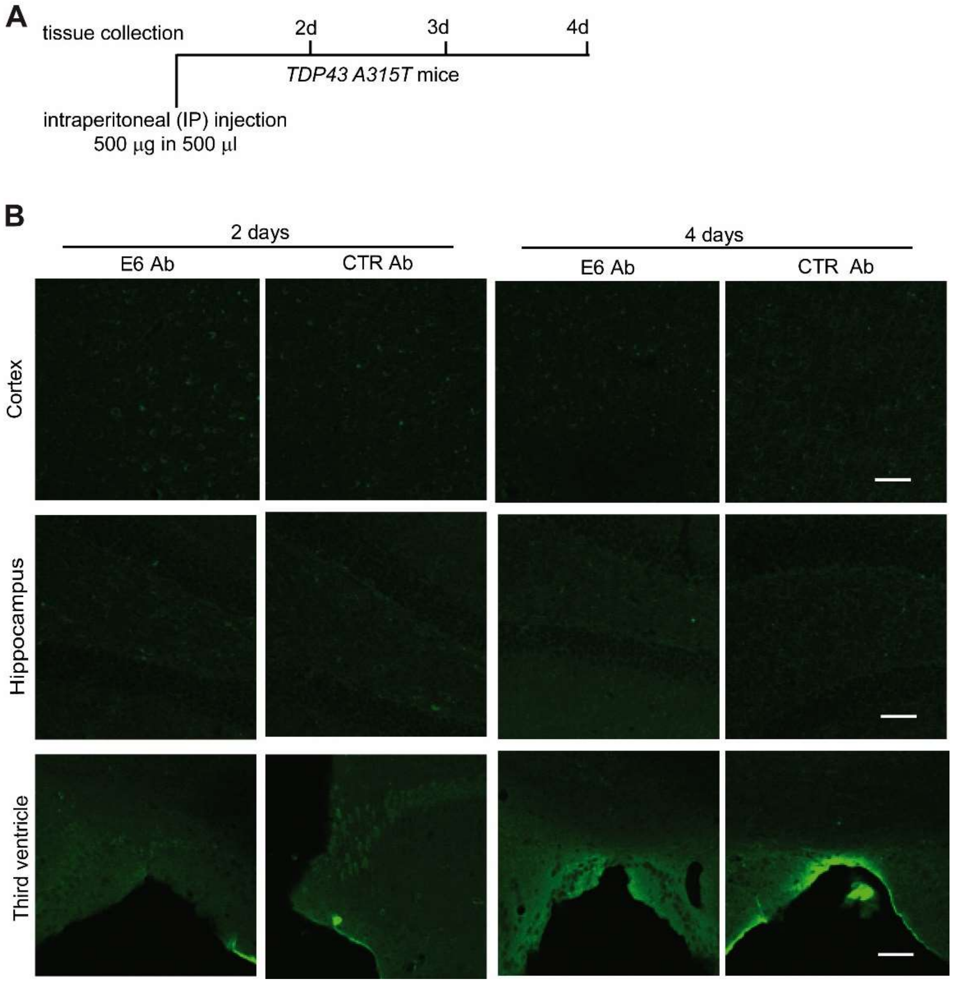

Supplementary Figure 5. Tissue and cellular distribution after intraperitoneal (IP) delivery. (**A**) Schematic representation of intraperitoneal delivery and time points of tissue collection. (**B**) Ab distribution after 2 and 4 days of from injection in coronal sections of brain. Signal represents mIgG2A (green). Scale bar =50 µm. One mouse per condition was used but results were confirmed in multiple sections from the areas of interest. Control antibody (clone 807.33).

Supplementary Figure 6

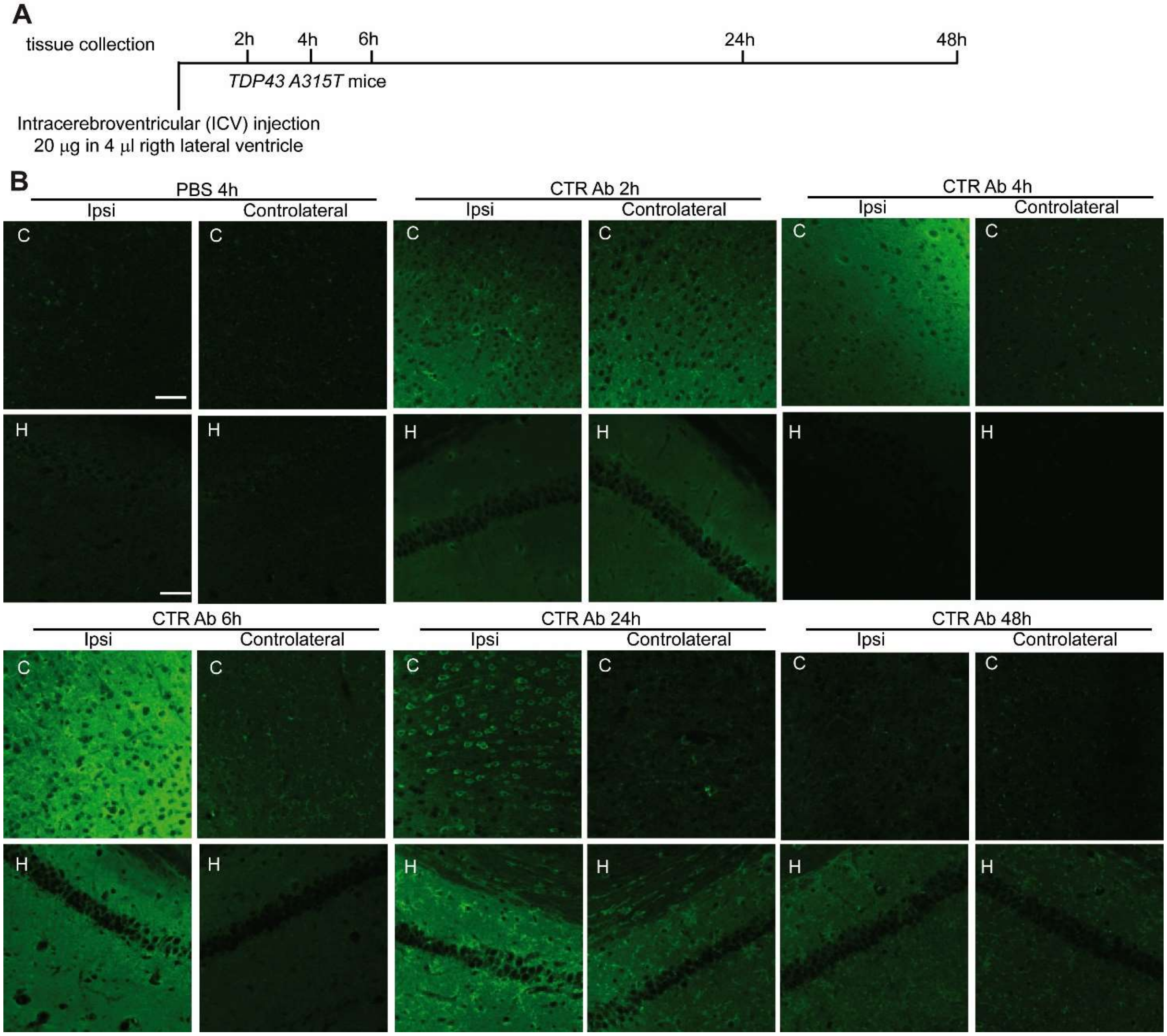

Supplementary Figure 6. Tissue and cellular distribution after intracerebroventricular (ICV) delivery. (**A**) Schematic representation of intracerebroventricular delivery and time points of tissue collection. (**B**) Control Ab (clone 807.33) distribution in coronal sections of cortex (C) and hippocampus (H). Ipsilateral and contralateral sections have been both considered. Signal represents mIgG2A (green). Scale bar =50 µm. One mouse per condition was used but results were confirmed in multiple sections from the areas of interest.

Supplementary Figure 7

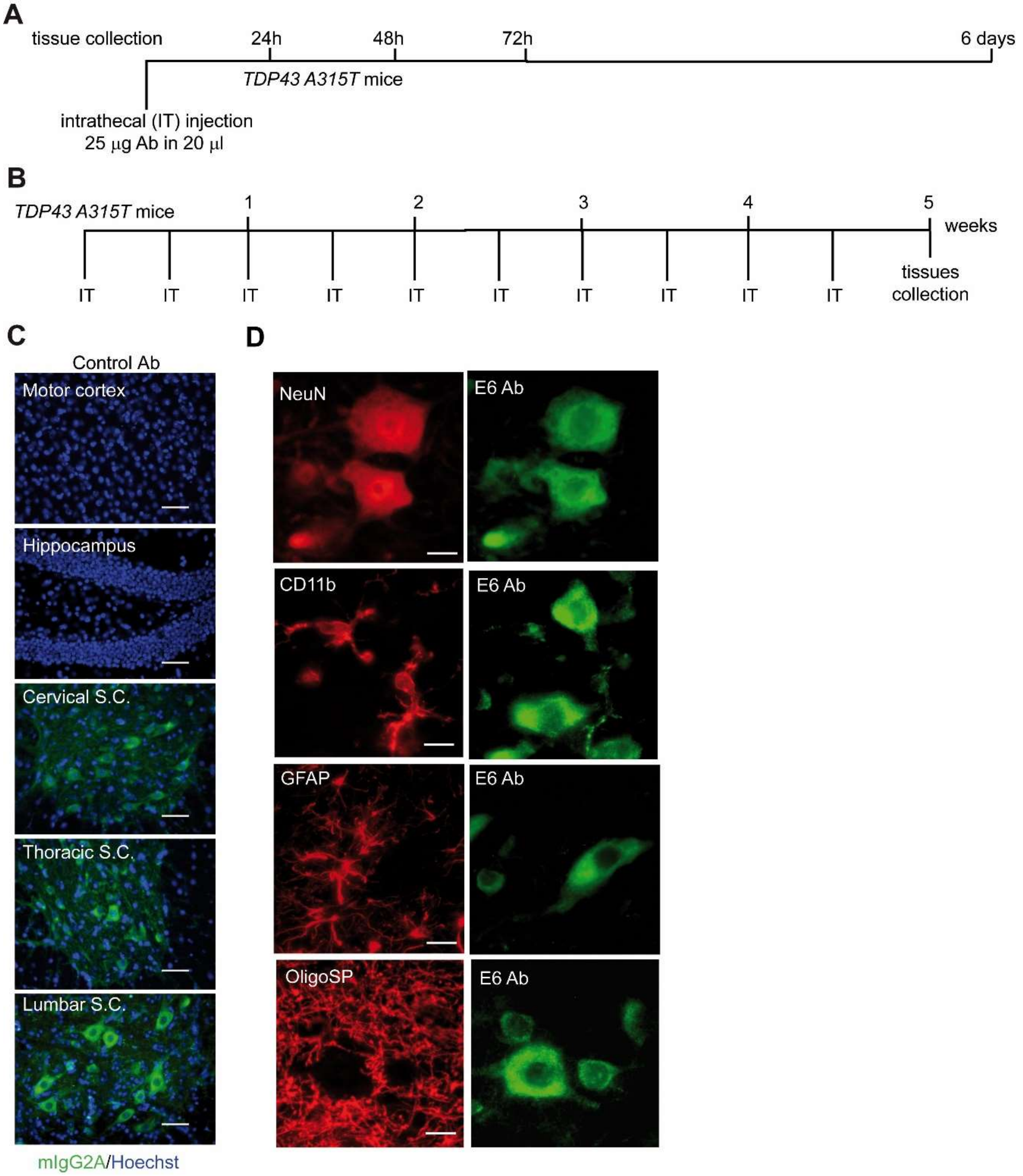

Supplementary Figure 7. Tissue and cellular distribution after intrathecal (IT) delivery. (**A**) Schematic representation of intrathecal delivery and time points of tissue collection. (**B**) Schematic representation of 5 weeks repeated intrathecal delivery in *TDP43A315T* mice. (**C**) Representative images of control antibody (mIgG2A staining, green merged with Hoechst, blue) distribution in the lumbar, thoracic and cervical regions of the spinal cord (S.C.) and brain (hippocampus and motor cortex) after 5 weeks of repeated intrathecal injections. Scale bar=50µm. **(D)** Representative high magnification images of immunofluorescence for E6 antibody (mIgG2A staining, green) and markers for neurons (NeuN, red), microglial cells (CD11B, red), astrocytes (GFAP, red) and oligodendrocytes (OligoSP, red) in ventral horns of lumbar spinal cord after 5 weeks of repeated injections. Merged channels are shown in figure 7D. Scale bar=20µm. Three mice per condition were used and results in panels C and D were confirmed in multiple sections from the areas of interest. Control antibody (clone 807.33).

Supplementary Figure 8

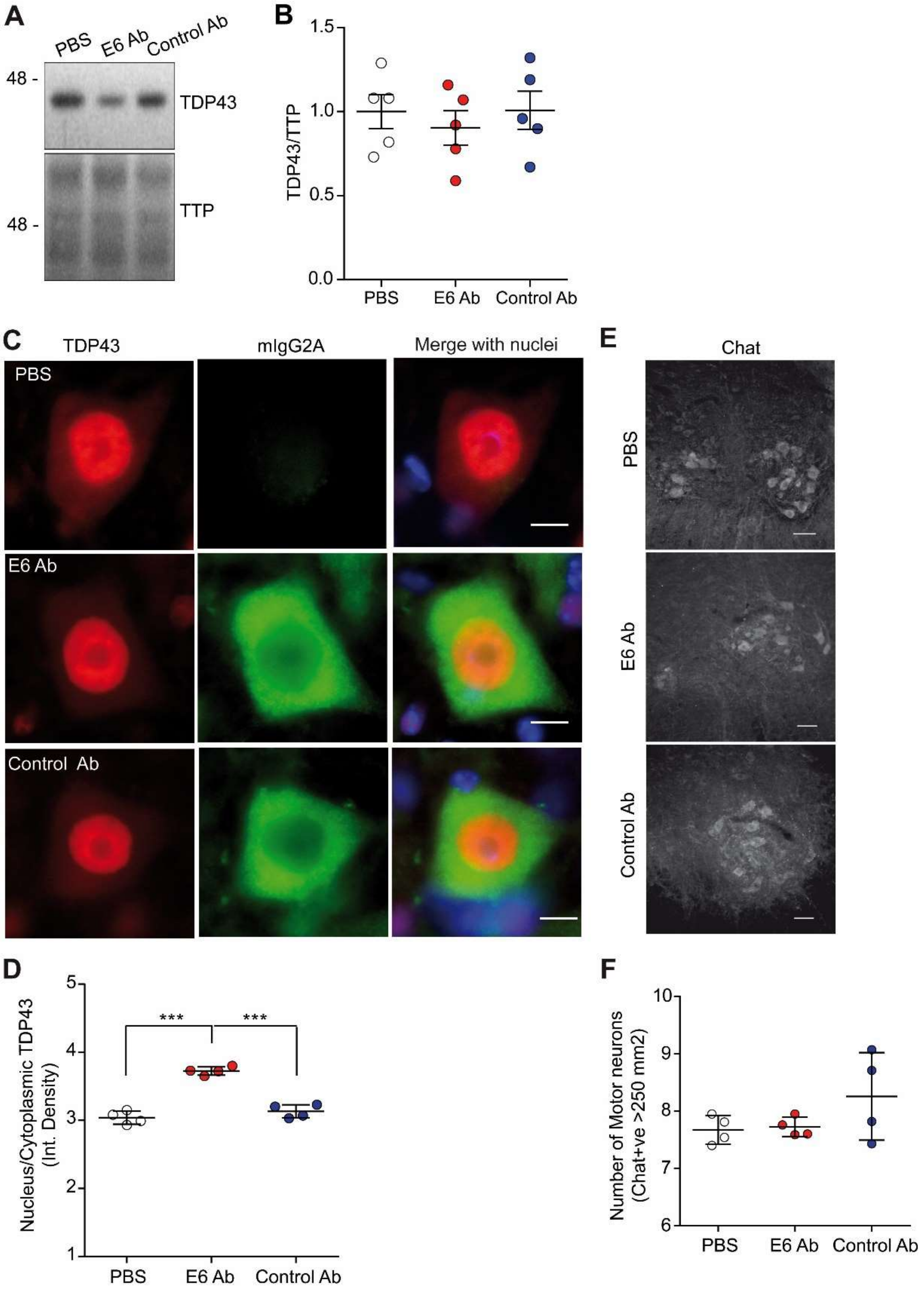

Supplementary Figure 8. Treatment with E6 antibody reduces cytoplasmic TDP43 in lumbar spinal cord neurons. (A) Representative western blot and relative quantification (**B**) of TDP43 performed on cytoplasmic fraction of lumbar spinal cord from treated mice. The C-Terminal anti-TDP43 antibody (Proteintech) was used, total transferred protein (TTP) were considered as loading reference. Data are mean±SEM (n=5 independent mice, dots), One-way Anova F2,12=0.3 P=0.07457. **(C)** Representative high magnification images of immunofluorescence for TDP43 (C-Terminal, Proteintech, red), mIgG2A antibody (green) and merge with nuclei (Hoechst, blue). Colorimetric heat map for TDP43 signal is shown in figure 8A. (**D**) Graph represents quantification of nuclear to cytoplasmic integrated density of TDP43 signal in single large neurons counted in

ventral horns of mice lumbar spinal cord. Data are mean±SEM (n=4 independent mice, dots), One-way Anova $F_{2,9}=73.42$ $P<0.0001$, *** $P<0.0001$ by Tukey's multiple comparison test. **(E)** Representative image and relative quantification (**F**) of Chat immunofluorescence staining in the anterior horn of lumbar spinal cords of treated mice for motor neurons counting. Scale bar 50 μm. Chat positive cells larger than 250 μm^2 were considered for analysis. Data are mean±SEM (n=4 independent mice, dots), One-way Anova $F_{2,9}=1.863$ $P=0.2104$. Control antibody (clone 807.33).

Supplementary Figure 9

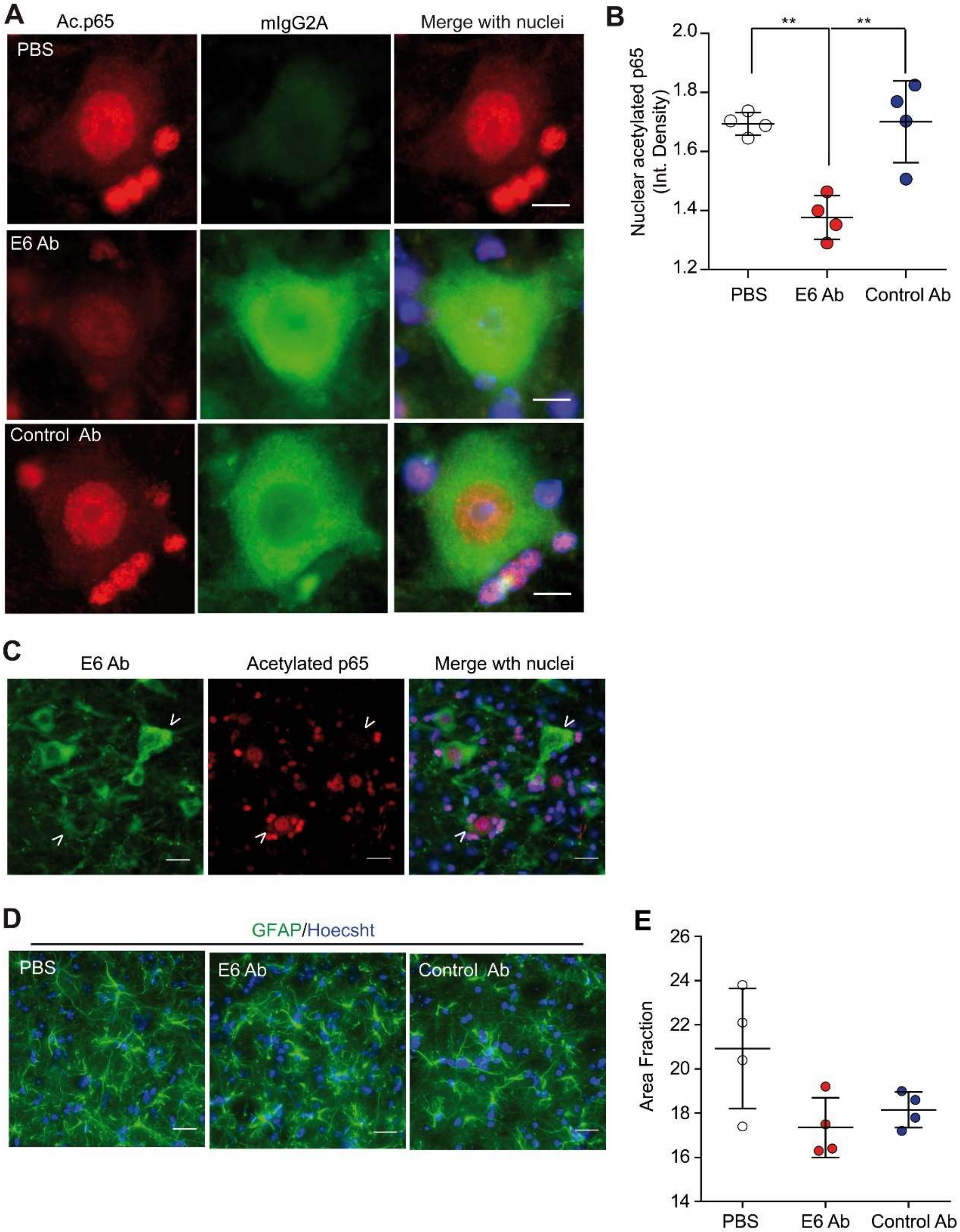

Supplementary Figure 9. Treatment with E6 antibody reduces nuclear p65 in lumbar spinal cord neurons. (A) Representative high magnification images of immunofluorescence for acetylated p65 (red), mIgG2A antibody (green) and merge with nuclei (Hoechst, blue). Colorimetric heat map for p65 signal is shown in figure 8C. Scale bar=10 μm. (**B**) Graph represents quantification of nuclear integrated density of p65 signal in single neurons counted in anterior horns lumbar spinal cord. Data are mean±SEM (n=4 independent mice, dots), One-way Anova F2,9=15.76 P=0.0011, ** P<0.01 by Tukey's multiple comparison test. (**C**) Representative images of immunofluorescence for acetylated p65 (red) and mIgG2A antibody (green), merge with nuclei is also shown. Arrow heads show two neurons where levels of nuclear p65 correlated with the amount of antibody uptaken by the cell. Scale bar=50 μm. Experiment was performed on four mice per condition. **(D)** Representative images of

immunofluorescence for astrocytic cells (GFAP, red) merged with nuclei (Hoechst, blue) performed on lumbar spinal cord of treated mice. Scale bar=20 μm. (**E**) Graphs represent quantification of percentage of area covered by GFAP signal (area fraction). Data are mean±SEM (n=4 independent mice, dots). Area Fraction One-way Anova $F_{2,9}=4.260$ $P=0.0499$. Control antibody (clone 807.33).

Supplementary methods

Cell cultures transfection and tests

HEK293 (human embryonic kidney cells, ATCC), Neuro2A cells (mouse neuroblastoma cells, ATCC) or BV2-*p65-luciferace* (mouse microglial cells) (1) were grown in Dulbecco's Modified Eagle Medium (DMEM, Gibco) with 10% foetal bovine serum (FBS, Gibco), 1% penicillin-streptomycin (Gibco) and 1% glutamine (Gibco) in a humidified atmosphere (95% air,5% CO2) at 37°C. Cells were transfected for 48h when at 70% confluence with human *Flag-TDP43WT* or human GFP-*mutantNLS-TDP43* (kindly provided by Dr. M. Urushitani) or *Tsg-101-mCherry* (2) plasmids or co-transfected with *GFP-mNLS-TDP43* and *mCherry-TRIM21* (Addgen #105516) or mock plasmid (pcDNA3) using Jet Prime (Polyplus) transfection reagents according to manufacturer's instructions.

Cells were treated with anti-TDP43 (E6) or control antibody (807.33) as stated in results or figure legends. BV2-*p65-luc* cells were treated for 4h with 500 ng/ml LPS (Sigma). To investigate the presence of the antibodies inside the cells, before homogenizations cells were abundantly washed with PBS for at least three times. To check for TDP43 degradation, after 24h from transfection, medium was replaced with Optimem (Gibco) containing 10 μg/ml cycloheximide (CHX) (Sigma). Cells where then treated for 24h with 10 μg/ml of antibodies and 0.5 μM of MG-132 (Sigma) or DMSO (dimethyl-sulfoxide, Sigma) when specified.

Luciferase activity was estimated using Bright-Glo Luciferase assay system (Promega) as previously described (3). Cell viability was assessed using Cell Titer 96 Aqueous One Solution Cell Proliferation assay (Promega) according to manufacturer's instructions. Luminescence and absorbance were read using an EnSpire 2300 Multilabel reader (Perkin Elmer).

Lysosomes were visualized by incubating cells for 1h at 37°C with Lysotracker Deep Red (Thermo Fisher) according to manufacturer's instructions before washing and fixing cells.

ELISA Array

ELISA were performed as previously described (1) using Elisa assay kit (Peprotech). Different concentration of recombinant TDP43 (home-made) (1) or BSA (BioBasic) were diluted in PBS, loaded on an Elisa assay plate and incubated overnight at room temperature. After washes, wells were incubated for 2h with PBS, 0.4 μg/ml of anti-RRM1 TDP43 (E6) or control (807.33) monoclonal antibodies or 0.4 μg/ml of monoclonal anti- human N-Terminal TDP43 (Abnova) diluted in PBS. Wells were then incubated 2h with goat anti-mouse HRP conjugated antibody (Jackson Immunoresearch) 1:10000. Chemiluminescence was read at 450 nm using an EnSpire 2300 Multilabel reader (Perkin Elmer).

Immunohistochemistry and Immunofluorescence

Formalin-fixed human tissue samples were embedded in paraffin, cut at 4 μm using a Leica RM125 microtome (Leica Biosystems, Wetzlar, Germany) and used for immunohistochemistry evaluations. Sections were collected on slides and immunostained using a fully automated IHC stainer Leica-BOND III (Leica Biosystems, Wetzlar, Germany). Finally, samples were analyzed under a Zeiss Axioscop40 microscope equipped with an Axio-cam MRC5 camera, using AxioVisio 4.6 software (Carl Zeiss, Oberkochen, Germany).

Human frontal cortex and lumbar spinal cord samples were cryo-sectioned at −20 °C into 5 μm thick sections using a Leica CM3050 S cryostat (Leica Biosystems). Frozen sections were air-dried for 45 minutes, washed three washes in PBS, blocked and permeabilized in 0.1% triton X-100 (Sigma) and 5 % bovine serum albumin (BSA) (Sigma-Aldrich) in PBS for 1 h at room temperature (RT) and incubated over night at 4 °C with primary antibodies (Supplementary Table 1) diluted in blocking solution. The next day, after washes, sections were incubated with appropriate Alexa Fluo-conjugated secondary antibodies (Thermo Fisher Scientific) diluted in blocking solution for 2 hours at RT. Finally, nuclei were counterstained with Hoechst 33342 (Thermo Fisher) diluted in PBS (1:200) for 5 minutes at RT.

Paraformaldehyde fixed mice lumbar spinal cords and brains were 25µm sectioned using a SM2000 R microtome (Leica Microsystems). Free-floating sections were washed in PBS, blocked and permeabilized in 0.1% Triton X-100 and 5% normal goat serum (NGS, Gibco) in PBS for 1h at room temperature and then incubated over night with primary antibodies (Supplementary Table 1) in blocking solution. The next day, after washes, sections were incubated with appropriate Alexa Fluor conjugated secondary antibodies (Invitrogen, Carlsbad, CA, USA, 1:1000) for 2 hours at room temperature. Nuclei were counterstained using Hoechst 33342 (Invitrogen) 1:10000 for 1min, and TrueBlack (Biotium) 1:20 for 2min was used to quench lipofuscin autofluorescence. Cells seeded in 24/48-well plates with cover glasses in the bottom. After transfections/treatments, cells were washed once in PBS and fixed with 4% paraformaldehyde for 15 min at room temperature. After three washes in PBS, cells were blocked and permeabilized in 0.1% triton X-100 and 5% bovine serum albumin in PBS for 1 h at room temperature. Cells were then washed and incubated over night at 4°C with primary antibodies (Supplementary Table 1) diluted in blocking solution. After washes in PBS, cells were incubated with appropriate Alexa Fluor conjugated secondary antibodies (Invitrogen, Carlsbad, CA, USA, 1:1000). Nuclei were counterstained with Hoechst 33342 (Thermo Fisher Scientific) 1:1000 in PBS.
Sections and cover glasses were mounted on slides (Fisher Scientific) using Fluoromount-G Buffer (Southern Biotech).

Protein extraction
Total proteins were obtained from cells or animal tissues using boiled SDS 1% as previously described (4). Briefly, samples were homogenized, sonicated, boiled 10 min at 99°C and cleared by centrifugation at 13000 rpm for 10 min. Cytoplasmic proteins were obtained from cells as previously described (5). Briefly, pellet of cells was lysed in RIPA-A (0.3% Triton X-100, 50mM Tris-HCl pH 7.4 and 1mM EDTA and protease inhibitors) and rotated at 4°C 30 min. Cytoplasm (supernatant) was obtained after centrifugation at 12000 x g 4°C for 10 min. Proteins were quantified by DC protein assay (Bio-Rad).

Dot blot and Western Blot
Dot blots were performed by loading different proteins on a PVDF membrane by vacuum filtration using a Dot-Blot apparatus (Bio-Rad). For Western blots, equal amount of proteins was resuspended in loading buffer supplemented with Dithiothreitol (DTT, Sigma) 1mM, boiled 5 min at 99°C and loaded on stain-free acrylamide gels (Bio-Rad) (6). Gels were UV-activated then transferred on PVDF membranes (Millipore). Membranes were washed with TBST (TBS, 0.1% Tween 20), blocked in 3% BSA prepared in TBST, incubated overnight with primary antibodies (Supplementary Table 1). Goat anti-mouse or anti-rabbit HRP (Jackson Immunoresearch) secondary antibodies were diluted 1:5000 in blocking solutions and incubated 2h RT, whereas anti-mouse-488 and anti-rabbit-555 Alexa Fluor conjugated secondary antibodies (Invitrogen) were diluted 1:1000 in TBST and incubated 1h RT in dark. ChemiDoc XRS (Bio-Rad) was used to acquire chemiluminescence, fluorescence, total transferred proteins (TTP) as stain free acquisition or Ponceau staining (Sigma). IRDye-conjugated (Li-Cor) secondary antibodies were used diluted 1:10000 in TBST and incubated 2h RT in the dark. Signal was acquired using the Li-Cor Sistem (Li-Cor Biotechnology). Densitometry was done by Image Lab (Bio-Rad) or Image Studio Lite 4.0 (Li-Cor) softwares.

Supplemetary Table 1. Antibodies used for western/dot blots or immunofluorescence.

Antigen	Antibody/clone	WB/DOT dilution	IF dilution	Company	Catalog number
Actin	mouse monoclonal/ clone C4	1 :10000		Millipore (Temecula CA, USA)	MAB1501
CD11b	rat monoclonal		1 :500	Bio-Rad	MCA711
Choline acetyltransferase (CHAT)	goat polyclonal		1 :500	Millipore	AB144
G1 protein of La Cross virus (CTR Ab)	IgG2A mouse monoclonal/ clone 807.33	0.02 µg/ml	1 :500	Medimabs (Montreal, QC, CA)	Not commercially available
Glial fibrillary acidic protein (GFAP)	rabbit polyclonal		1:1000	Dako (Santa Clara, CA, USA)	Z0334
Iba1	rabbit polyclonal		1:500	Wako Chemicals (Richmond, VA, USA)	019-19741
Mouse IgG2A	goat polyclonal (alexa-488 conjugated)		1:4000	Invitrogen (Carl sbad, CA, USA)	A-21131
Mouse IgG2A	goat polyclonal (HRP conjugated)	1 :1000		Invitrogen (Carl sbad, CA, USA)	M32207
NeuN	rabbit monoclonal/ clone D3S3I		1:500	Cell Signaling Technologies	D3531
NF-KB p65 (acetyl K310)	rabbit polyclonal		1:1000	Abcam (Cambridge, UK)	ab19870
RRM1 TDP43 (E6)	IgG2A mouse monoclonal/ clone 52E6	0.02 µg/ml	1 :500	Medimabs (Montreal, QC, CA)	Not commercially available
TDP-43 N-Term	mouse monoclonal/ clone 2E2-D3	1:1000		Abnova (Taipei City, Taiwan)	H00023435-M01
TDP43 C-Term	rabbit polyclonal	1:5000	1 :1000	Proteintech (Chicago, IL, USA)	12892-2-AP
TRIM21	rabbit polyclonal		1 :1000	Proteintech (Chicago, IL, USA)	12108-1-AP

References of supplementary material

1. Pozzi S et al. Virus-mediated delivery of antibody targeting TAR DNA-binding protein-43 mitigates associated neuropathology. *J. Clin. Invest.* 2019;129(4):1581–1595.

2. Iguchi Y et al. Exosome secretion is a key pathway for clearance of pathological TDP-43. *Brain* 2016;139(12):3187–3201.

3. Dutta K, Patel P, Rahimian R, Phaneuf D, Julien JP. Withania somnifera Reverses Transactive Response DNA Binding Protein 43 Proteinopathy in a Mouse Model of Amyotrophic Lateral Sclerosis/Frontotemporal Lobar Degeneration. *Neurotherapeutics* 2017;14(2):447–462.

4. Pozzi S, Thammisetty SS, Julien JP. Chronic Administration of Pimozide Fails to Attenuate Motor and Pathological Deficits in Two Mouse Models of Amyotrophic Lateral Sclerosis. *Neurotherapeutics* 2018;15(3):715–727.

5. Lauranzano E et al. Peptidylprolyl isomerase A governs TARDBP function and assembly in heterogeneous nuclear ribonucleoprotein complexes. *Brain* 2015;138(4):974–991.

6. Taylor SC, Berkelman T, Yadav G, Hammond M. A defined methodology for reliable quantification of western blot data. *Mol. Biotechnol.* 2013;55(3):217–226.
